# Impact of PIK3CA Mutations on the Clinical Benefits of CDK 4/6 Inhibitors in HR+/HER2- Advanced Breast Cancer: An Updated Pairwise and Network Meta-Analysis

**DOI:** 10.7150/jca.111362

**Published:** 2025-07-04

**Authors:** Sheng-Fan Wang, Chian-Ying Chou, Yi-Wen Chao, Tzu-Kuan Chan, Wan-Yu Yeh, Chern-En Chiang, Chen-Huan Chen, Hsin-Chen Lee, Ling-Ming Tseng, Yuh-Lih Chang, Hao-Min Cheng, Chun-Yu Liu

**Affiliations:** 1 Department of Pharmacy, Taipei Veterans General Hospital, No. 201, Sec. 2, Shipai Rd., Beitou, Taipei 112, Taiwan.; 2 Department of Clinical Pharmacy, School of Pharmacy, Taipei Medical University, No. 250, Wuxing St., Xinyi, Taipei 110, Taiwan.; 3 Department and Institute of Pharmacology, College of Medicine, National Yang-Ming Chiao-Tung University, No. 155, Sec. 2, Linong St., Beitou, Taipei 112, Taiwan.; 4 Institute of Biopharmaceutical Sciences, College of Pharmaceutical Sciences, National Yang-Ming Chiao-Tung University, Taipei, Taiwan, No. 155, Sec. 2, Linong St., Beitou, Taipei 112, Taiwan.; 5 Department of Pharmacy, College of Pharmaceutical Sciences, National Yang-Ming Chiao-Tung University, No. 155, Sec. 2, Li-Nong St., Beitou, Taipei 112, Taiwan.; 6 Center for Evidence-based Medicine, Taipei Veterans General Hospital, No. 201, Sec. 2, Shipai Rd., Beitou, Taipei 112, Taiwan.; 7 Department of Medical Education, Taipei Veterans General Hospital, No. 201, Sec. 2, Shipai Rd., Beitou, Taipei 112, Taiwan.; 8 School of Medicine, College of Medicine, National Yang-Ming Chiao-Tung University, No. 155, Sec. 2, Linong St., Beitou, Taipei 112, Taiwan.; 9 General Clinical Research Center, Taipei Veterans General Hospital, No. 201, Sec. 2, Shipai Rd., Beitou, Taipei 112, Taiwan.; 10 Institute of Public Health and Community Medicine Research Center, National Yang-Ming Chiao-Tung University School of Medicine, No. 155, Sec. 2, Linong St., Beitou, Taipei 112, Taiwan.; 11 Department of Medical Research, Taipei Veterans General Hospital, No. 201, Sec. 2, Shipai Rd., Beitou, Taipei 112, Taiwan.; 12 Comprehensive Breast Health Center, Department of Surgery, Taipei Veterans General Hospital, No. 201, Sec. 2, Shipai Rd., Beitou, Taipei 112, Taiwan.; 13 Division of Medical Oncology, Department of Oncology, Taipei Veterans General Hospital, No. 201, Sec. 2, Shipai Rd., Beitou, Taipei 112, Taiwan.

**Keywords:** cyclin-dependent kinase 4/6, hormone receptor-positive, human epidermal growth factor receptor-2 negative, advanced breast cancer, *PIK3CA*

## Abstract

**Background:** Cyclin-dependent kinase 4/6 inhibitors (CDK4/6i) have revolutionized the treatment of hormone receptor-positive (HR+) and human epidermal growth factor receptor-2 negative (HER2-) advanced breast cancer. However, identifying reliable biomarkers and determining overall survival (OS) outcomes for CDK4/6i remains challenging.

**Methods:** We conducted a systematic review and updated pairwise meta-analysis of randomized controlled trials to evaluate the clinical benefits and biomarker interactions of CDK4/6i in HR+ and HER2- advanced breast cancer. Hazard ratio (HR) and 95% confidence interval (CI) were calculated for progression-free survival (PFS) and OS across different clinical settings. Additionally, a network meta-analysis was performed to assess the comparative efficacy of different CDK4/6i in specific populations using ranking probabilities.

**Results:** CDK4/6i significantly improved PFS (HR 0.55, 95% CI 0.52-0.59) and OS (HR 0.80, 95% CI 0.74-0.86) in patients with HR+/HER2- advanced breast cancer. Sensitivity analyses confirmed the robustness of these findings. Subgroup and meta-regression analyses demonstrated consistent clinical benefits across different lines of therapy, endocrine therapy categories, patient characteristics, and follow-up durations. However, *PIK3CA* mutation status emerged as a potential CDK4/6i efficacy modifier, particularly among patients who were endocrine therapy-naïve for advanced disease (First-line treatment: *p* for interaction = 0.03; received prior treatment, *p* = 0.68). The network meta-analysis suggested comparable overall efficacy among CDK4/6i. However, ribociclib may offer a slight OS advantage over palbociclib in first-line treatment, with ranking probabilities varying by specific clinical settings.

**Conclusions:** This updated meta-analysis further validates the OS benefit of CDK4/6i in HR+/HER2- advanced breast cancer. The influence of *PIK3CA* mutation status on CDK4/6i efficacy appears more pronounced in endocrine therapy-naïve patients rather than those receiving later-line therapy. While currently approved CDK4/6 inhibitors exhibit similar efficacy overall, their ranking probabilities vary depending on individual clinical contexts. These findings highlight the need for further investigation into the modifying effects of *PIK3CA* status and specific CDK4/6i to optimize treatment strategies in HR+/HER2- advanced breast cancer.

## Introduction

Breast cancer is the most common cancer among women 1. Gene expression profiling has identified distinct molecular subtypes of breast cancer into luminal A, luminal B, human epidermal growth factor receptor-2 (HER2)-enriched, and basal-like. Hormone receptor-positive (HR+)/HER2-negative (HER2-) breast cancers are the most common subtype 2.

Mitogenic responses, such as estrogen signaling, initiate events that activate genes necessary for the cell cycle process. Cyclin D, a protein that increases in response to signaling, plays a vital role in this process and binds to cyclin-dependent kinase (CDK) 4 or 6, forming a complex that phosphorylates Rb and activates the cell cycle. The cyclin D-CDK4/6-p16-Rb pathway is commonly dysregulated in cancer and is a promising target against cancer 3. In breast cancer patients with HR+ tumors, the activation of the CDK4/6 pathway has been identified as a contributing factor to resistance against endocrine therapy 4. Specific inhibitors against CDK4/6 were recently introduced in cancer therapy 5. For patients with HR+ metastatic breast cancer, combining CDK 4/6 inhibitors with endocrine therapy is recommended 6. However, the lack of direct comparative trials between CDK 4/6 inhibitors has led to ongoing controversy over which inhibitor should be prioritized.

Despite the proven efficacy of CDK4/6 inhibitors, it has been observed that a subset of patients may exhibit intrinsic or acquired resistance 7. Although considerable effort has been made to assess potential resistance mechanisms, the available evidence is primarily derived from preclinical studies, with limited clinical evidence of acquired genomic alterations linked to resistance 8. The phosphatidylinositol-4,5-bisphosphate 3-kinase catalytic subunit alpha (*PIK3CA*) gene, which encodes the p110α isoform of phosphatidylinositol 3-kinase (PI3K), is frequently mutated (approximately 40%) in the HR+/HER2- subgroup 9. Dysregulation of the PI3K pathway is associated with tumorigenesis and resistance, highlighting the prognostic relevance of *PIK3CA* mutations in breast cancer patients 10. Recently, the aggregated data from multiple studies have demonstrated a correlation between *PIK3CA* mutation and shortened progression-free survival (PFS) and overall survival (OS) in patients with HR+/HER2-metastatic breast cancer, indicating that *PIK3CA* mutations possess negative prognostic value for these patients 11.

Pooled circulating tumor DNA analysis from the MONALEESA phase III advanced breast cancer trials showed that certain genomic alterations, such as Erb-B2 receptor tyrosine kinase 2 (HER2), might be associated with favorable PFS when treated with ribociclib compared to placebo 9. In contrast, the clinical significance of *PIK3CA* alteration remains limited 9. The PALOMA-2 trial found that palbociclib combined with fulvestrant provided a more extended period without cancer progression, regardless of *PIK3CA* status 12. Similarly, the combination of abemaciclib and fulvestrant effectively treated breast cancer patients, irrespective of their *PIK3CA* status, as observed in the MONARCH-2 trial 13. However, an interaction effect of *PIK3CA* alteration was noted in the MONARCH-3 trial 14.

Several lines of evidence have proposed the clinical benefits of CDK4/6 inhibitors in advanced breast cancer. However, the statistical significance of OS differences between CDK4/6 inhibitors remains controversial and uncertain 15. Recently, several new CDK4/6 inhibitors, such as lerociclib and dalpiciclib, have been introduced 16, 17, and updated clinical benefits of CDK4/6 inhibitors have been released 9, 18, 19. In this study, we further investigated the clinical benefits of CDK4/6 inhibitors on HR+ and HER2- advanced breast cancer using the latest reports to evaluate long-term clinical benefits and study the impact of biomarkers on the efficacy of CDK4/6 inhibitors, as well as compare the effectiveness of different CDK4/6 inhibitors in specific populations.

## Materials and Methods

### Search method and selection criteria

This study employed the PICO framework (Problem/population: HR+/HER2- advanced breast cancer; Intervention: CDK4/6 inhibitor; Comparison: Placebo; Outcome: PFS/OS). The research methodology included a systematic review, pairwise, and network meta-analysis to derive insightful results. We conducted searches in PubMed/PubMed Central, Embase, Cochrane Library, Web of Science, and the Google Scholar online source without language restrictions up to April 15, 2025. The search terms (field for clinical trial) used were modified according to the previous study 20: """breast cancer"" OR ""breast cancers"" OR ""breast neoplasm"" OR ""breast tumor"" OR ""breast malignancy"" OR ""breast carcinoma"" OR ""breast adenocarcinoma"" AND ""CDK4/6 inhibitor"" OR ""CDK4/6 inhibitors"" OR ""abemaciclib"" OR ""palbociclib"" OR ""ribociclib"" OR ""CDK inhibitor"" OR ""CDK inhibitors"" AND ""survival""" (To ensure a comprehensive and rigorous search aligned with our research focus, we additionally included the terms “abemaciclib,” “ribociclib,” “CDK4/6 inhibitor(s),” and “CDK inhibitor(s),” while excluding other targeted therapies and chemotherapies). We compiled available data and selected the most recent studies if multiple publications reported on the same clinical trial. This study adhered to the Preferred Reporting of Systematic Reviews and Meta-Analyses (PRISMA) extension statement for reporting systematic reviews incorporating network meta-analyses of health care interventions 21 (Checklist for network meta-analysis provided in Supplementary Table 1). Studies were included if they met the following criteria: (1) Focused on patients with HR+/HER2- advanced breast cancer, (2) randomized controlled trial (RCT), (3) compared CDK4/6 inhibitors to placebo or other CDK4/6 inhibitors, (4) de novo CDK4/6 inhibitors intervention, and (5) reported survival outcomes. The following categories were excluded: non-CDK4/6 inhibitors, estrogen receptor (ER)-negative breast cancer, non-RCT, non-breast cancer, comparisons other than placebo, not de novo CDK4/6 inhibitor, early-stage breast cancer, HER2-positive breast cancer, lacking survival outcomes or non-retrievable.

### Data collection and quality assessment

We used a standardized Microsoft Excel spreadsheet to document data from included studies. Extracted data included the following information: trial name, publication year, journal name, study design, number of patients, lines of therapy, category of endocrine therapy, biomarker status, follow-up time, median age, percentage of the white ethnic group, ECOG score = 0, previous chemotherapy or endocrine therapy, metastatic sites ≥ 3, and visceral metastases, PFS, and OS (Supplementary data).

The Cochrane tool (Risk of Bias 2) was employed to assess the risk of bias and quality in included RCTs 22, 23. Each assessment was classified as having a high, medium, or low risk of bias. The systematic review, which included a meta-analysis, examined key aspects, such as attrition bias, detection bias, performance bias, reporting bias, selection bias, and others. The systematic review with network meta-analysis evaluated several domains, including deviations from intended interventions, missing outcome data, outcome measurement, randomization process, selection of the reported result, and overall bias. The eligibility of reference and risk of bias in included studies were independently evaluated by two authors, S.F. Wang and Y.W. Chao. Discrepancies were resolved by consulting a third author, H.M. Cheng. This study was registered with PROSPERO, registration number CRD42024531849.

### Outcomes

The primary objective was to assess the clinical benefits of CDK4/6 inhibitors on PFS and OS. Subgroup analysis examined the impact of various factors, such as lines of therapy, category of endocrine therapy, and biomarker interaction, including *PIK3CA*, tumor protein p53 (*TP53*), and estrogen receptor 1 (*ESR1*) status. Meta-regression analysis evaluated the effects of follow-up time, median age, and percentage of white ethnic group, ECOG score = 0, previous chemotherapy or endocrine therapy, metastatic sites ≥3, and visceral metastases on PFS and OS. Sensitivity analysis for primary outcomes was conducted by excluding trials categorized as open-label RCT, such as PALOMA-1 RCT.

### Statistical analyses

The study computed hazard ratio (HR), risk ratio (RR), and 95% confidence interval (CI). The random effects model was chosen as the primary approach for comparison due to varying follow-up duration, CDK4/6 inhibitors, and combination with different endocrine therapies in RCTs. A significant threshold of *p* < 0.05 was set. Meta-analysis was performed using RevMan software 5.4, while meta-regression was conducted using Comprehensive Meta-Analysis V3 software. Post-hoc power tests with dmetar: Companion R Package were used to calculate the power analysis of primary outcomes as in the previous study 24. Statistical measures for heterogeneity included I², tau^2^, and Cochran's Q test, with significant heterogeneity defined as *p* < 0.10.

Confidence in Network Meta-Analysis (CINeMA) 25 was used to perform network meta-analysis. Ranking probability was determined by calculating P-scores based on network point estimates and standard errors using the netmeta package in R version 4.4.1. P-scores indicate the average level of certainty regarding the superior clinical benefit of treatments, rated on a scale from 1 (best) to 0 (worst). The netmeta package in R version 4.4.1 examined the assumptions of network meta-analysis, specifically homogeneity and consistency (inconsistency was not evaluated due to the lack of direct comparison between different CDK4/6 inhibitors). The similarity of baseline patient characteristics across various RCTs was assessed in Supplementary Fig. 1. The network diagram included nodes representing different CDK4/6 inhibitors and lines showing direct comparisons between pairs of interventions. Node magnitude in the network diagram represents the number of studies, the color indicates the level of bias, and the edge width represents the size of interventions. The absence of lines between nodes indicates a lack of studies comparing interventions between those nodes.

## Results

### Searching process, features of included studies, and design of analysis

**Figure 1:**
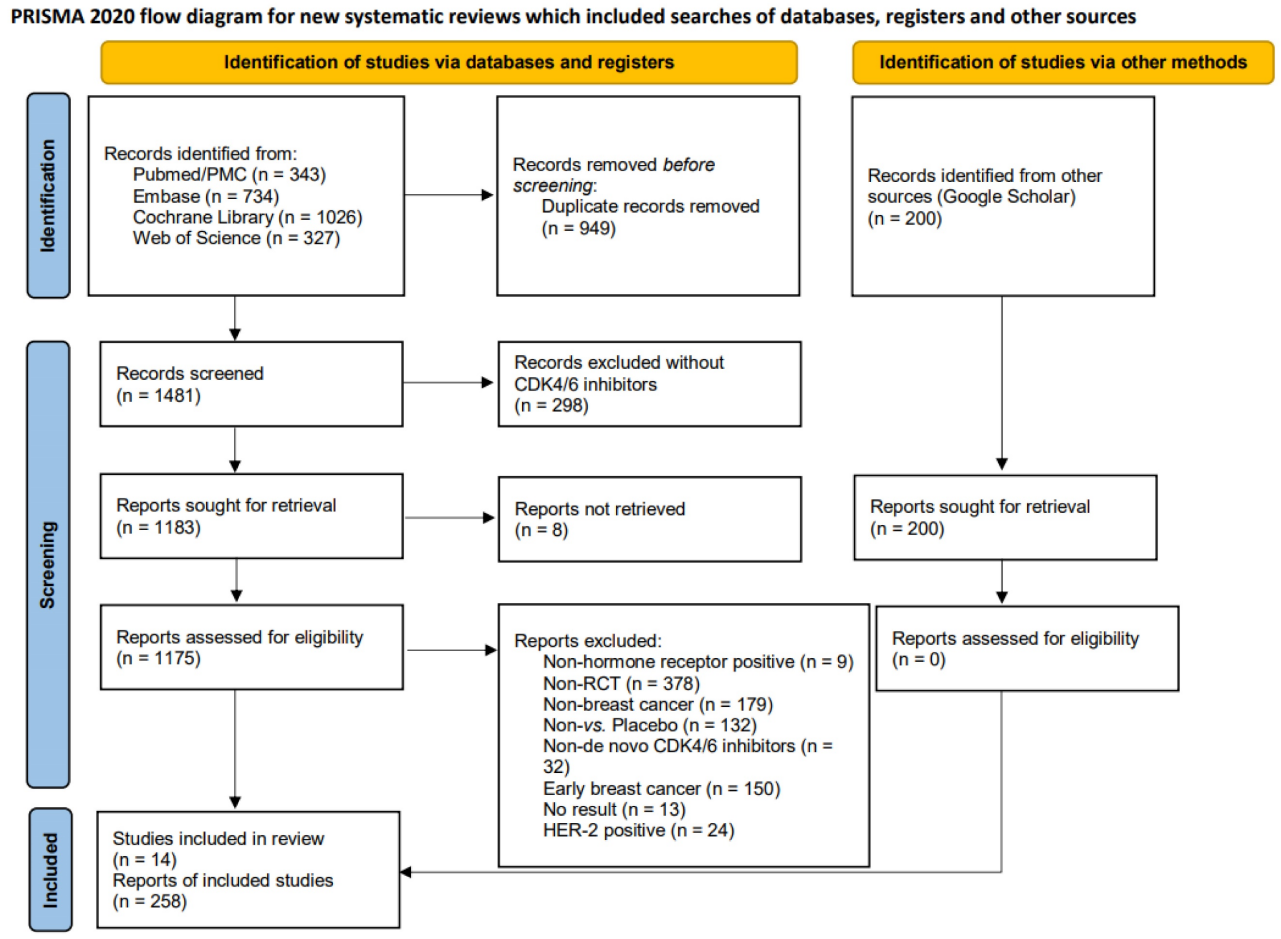
** The PRISMA 2020 flow diagram for this study.** This figure illustrates the study identification, screening, and selection process of clinical trials included in this research.

Throughout the search process, we identified a total of 2,430 publications from four databases: PubMed/PubMed Central (n = 343), Embase (n = 734), Cochrane Library (n = 1026), and the Web of Science (n = 327). In addition, 200 records were screened from Google Scholar. Fig. 1 illustrates the distribution of eligible publications across these databases. The preliminary step to exclude duplicate records (n = 949) was carried out using EndNote software. Subsequently, the remaining records underwent a rigorous screening process, which excluded any reports that did not involve CDK4/6 inhibitors (n = 298). Specifically, we excluded those records that were inaccessible (n = 8), unsuitable patient populations [i.e., non-HR+ breast cancer (n= 9), non-RCT (n = 378), non-breast cancer (n = 179), early breast cancer (n = 150), and HER2-positive breast cancer (n = 24)], lacked appropriate interventions [non-de novo CDK4/6 inhibitors (n = 32) and non *vs.* placebo (n = 132)], or lacked survival outcomes (n = 13). We also evaluated the Google Scholar online source. Finally, we included 258 reports, encompassing fourteen RCTs 16, 17, 19, 26-36, including DAWNA-2 16, LEONARDA-1 17, NCCH1607/PATHWAY 19, FLIPPER 26, MONALEESA-2 27, MONALEESA-3 28, MONALEESA-7 29, MONARCH-2 30, MONARCH-3 31, MONARCHplus 32, PALOMA-1 33, PALOMA-2 34, PALOMA-3 35, and PALOMA-4 36 (Baseline information of included trials is provided in Supplementary Table 2).

Most RCTs were designed as Phase III, except for the FLIPPER and PALOMA-1 Phase II clinical trials. Most RCTs were at least double-blinded except for the PALOMA-1 open-label trial. Patients in the DAWNA-2, FLIPPER, MONALESSA-2, MONALEESA-7, MONARCH-3, PALOMA-1, PALOMA-2, and PALOMA-4 RCTs had received no prior treatment for advanced disease (1^st^ line therapy for advanced disease). In contrast, patients in the LEONARDA-1, MONARCH-2, and PALOMA-3 RCTs had progressed after prior endocrine therapy (≥2^nd^ line therapy for advanced disease). The MONALESSA-3, MONARCHplus (Cohort-A for 1^st^ line and Cohort-B for ≥2^nd^ line), and NCCH1607/PATHWAY trials were designed for mixed-line therapy for advanced disease. All CDK4/6 inhibitor interventions were combined with endocrine therapy. In the NCCH1607/PATHWAY trial, tamoxifen was incorporated, whereas the DAWNA-2, MONALEESA-2, MONARCH-3, PALOMA-1, PALOMA-2, and PALOMA-4 trials adapted aromatase inhibitors. Fulvestrant was used in the FLIPPER, LEONARDA-1, MONALEESA-3, MONARCH-2, and PALOMA-3 trials. Some RCTs involved mixed endocrine therapy, such as MONALEESA-7 (tamoxifen or aromatase inhibitors) and MONARCHplus (Cohort A: aromatase inhibitor; Cohort B: fulvestrant). Specific CDK4/6 inhibitors were used in individual RCTs: dalpiciclib for DAWNA-2, palbociclib for FLIPPER, NCCH1607/PATHWAY, PALOMA-1, PALOMA-2, PALOMA-3, and PALOMA-4, lerociclib for LEONARDA-1, ribociclib for MONALEESA-2, MONALEESA-3, and MONALEESA-7, and abemaciclib for MONARCH-2, MONARCH-3, and MONARCHplus.

To better understand the clinical benefits of CDK4/6 inhibitors in patients with advanced breast cancer who are HR+ and HER2-, we conducted a meta-analysis comparing pooled data on the use of CDK4/6 inhibitors versus placebo from the most recent reports. Subsequently, the studies were further sub-grouped according to different lines of therapy, types of endocrine therapy, and biomarker status. Additionally, we utilized network meta-analysis to indirectly compare the clinical benefits of various CDK4/6 inhibitors.

### Meta-analysis for the effects of CDK4/6 inhibitors on PFS

**Figure 2:**
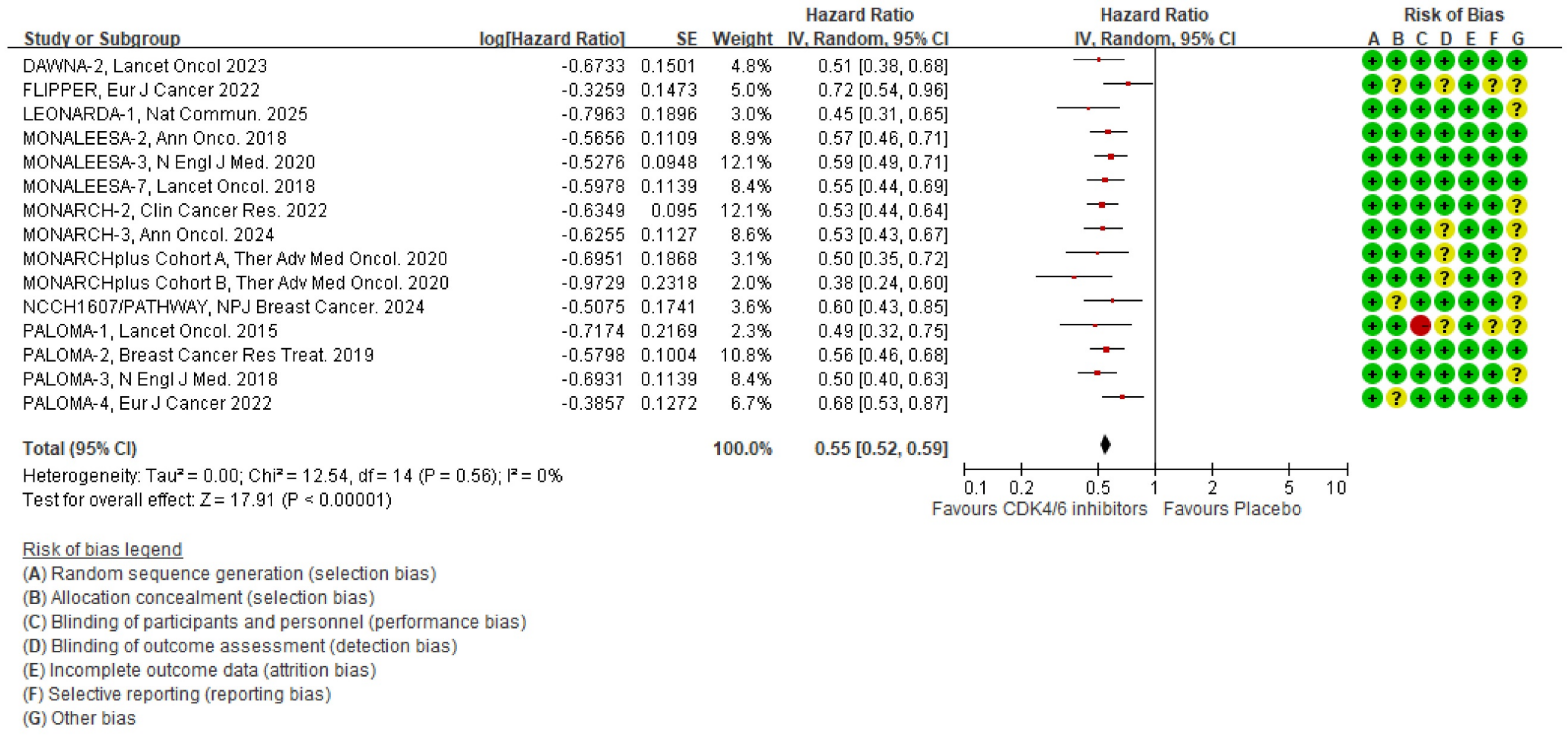
** Pooled meta-analysis of the effects of CDK4/6 inhibitors on PFS.** The forest plot shows the hazard ratios of CDK4/6 inhibitors versus placebo on PFS and the risk of bias for the included clinical trials. Colors represent the risk of bias: red indicates high risk, yellow indicates unknown risk, and green indicates low risk.

Based on the latest PFS reports 13, 16, 17, 19, 26, 29, 31-33, 36-40, we analyzed 14 RCTs that examined the impact of CDK4/6 inhibitors on PFS. The results showed that the CDK4/6 inhibitors significantly reduced the HR, as shown in Fig. 2 [HR 0.55 (95% CI 0.52-0.59), *p* < 0.00001]. The heterogeneity test for this outcome was insignificant (I^2^ = 0%, tau^2^ = 0, Q =12.54, *p* = 0.56), and the post-hoc power test showed 100 % effectiveness.

**Figure 3:**
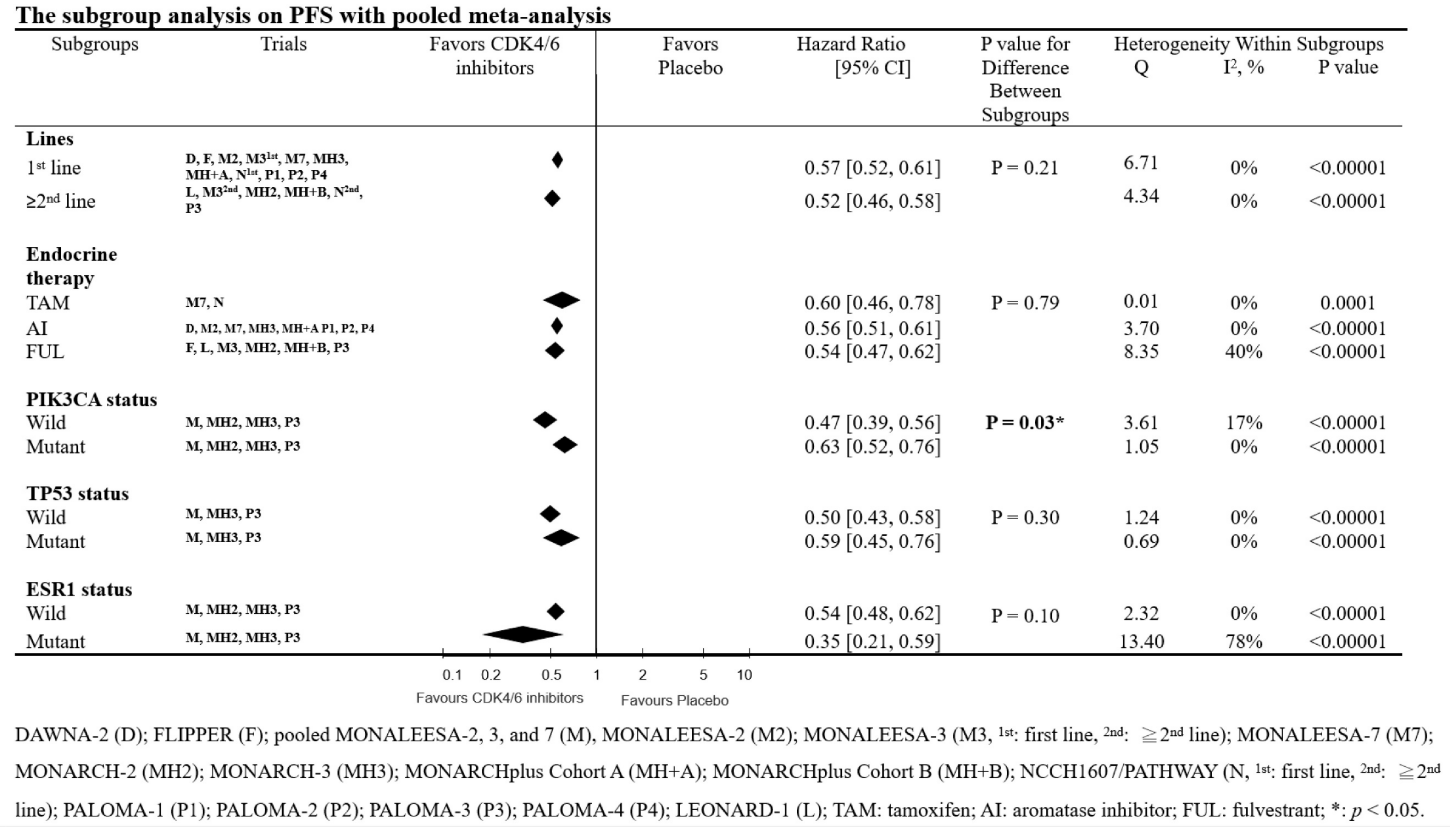
** Subgroup analysis of the effects of CDK4/6 inhibitors on PFS with pooled meta-analysis.** This forest plot presents the hazard ratios of CDK4/6 inhibitors versus placebo on PFS across different subgroups.

We conducted a subgroup analysis to examine the interactions of biomarkers (such as *PIK3CA*, *TP53*, and *ESR1*), different lines of therapy, and the category of combined endocrine therapy on the effectiveness of CDK4/6 inhibitors. For this purpose, we used the genomic analysis reports of the RCTs 9, 12-14. The subgroup analyses consistently demonstrated a PFS clinical benefit of CDK4/6 inhibitors (Fig. 3). In the subgroup analysis, we found that the differences between lines of therapy, types of endocrine therapy, and *TP53* & *ESR1* gene interaction were not significant (lines of therapy, *p* = 0.21; types of endocrine therapy, *p* = 0.79; *TP53* & *ESR1* status interaction, *p* = 0.30 and 0.10, respectively). However, we observed a significant difference between *PIK3CA* wild-type and mutant breast cancer patients (*p* = 0.03).

To comprehensively understand the potential factors influencing the clinical benefit of CDK4/6 inhibitors on PFS, we conducted a meta-regression analysis with demographic and clinical characteristics, including follow-up time, median age, and percentage of white ethnic group, ECOG score = 0, previous chemotherapy or endocrine therapy, metastatic sites ≥ 3, and visceral metastases. Our analysis revealed that none of these factors statistically impacted PFS (Table 1).

### The effect of *PIK3CA* status on the PFS clinical benefit of CDK4/6 inhibitors

**Figure 4:**
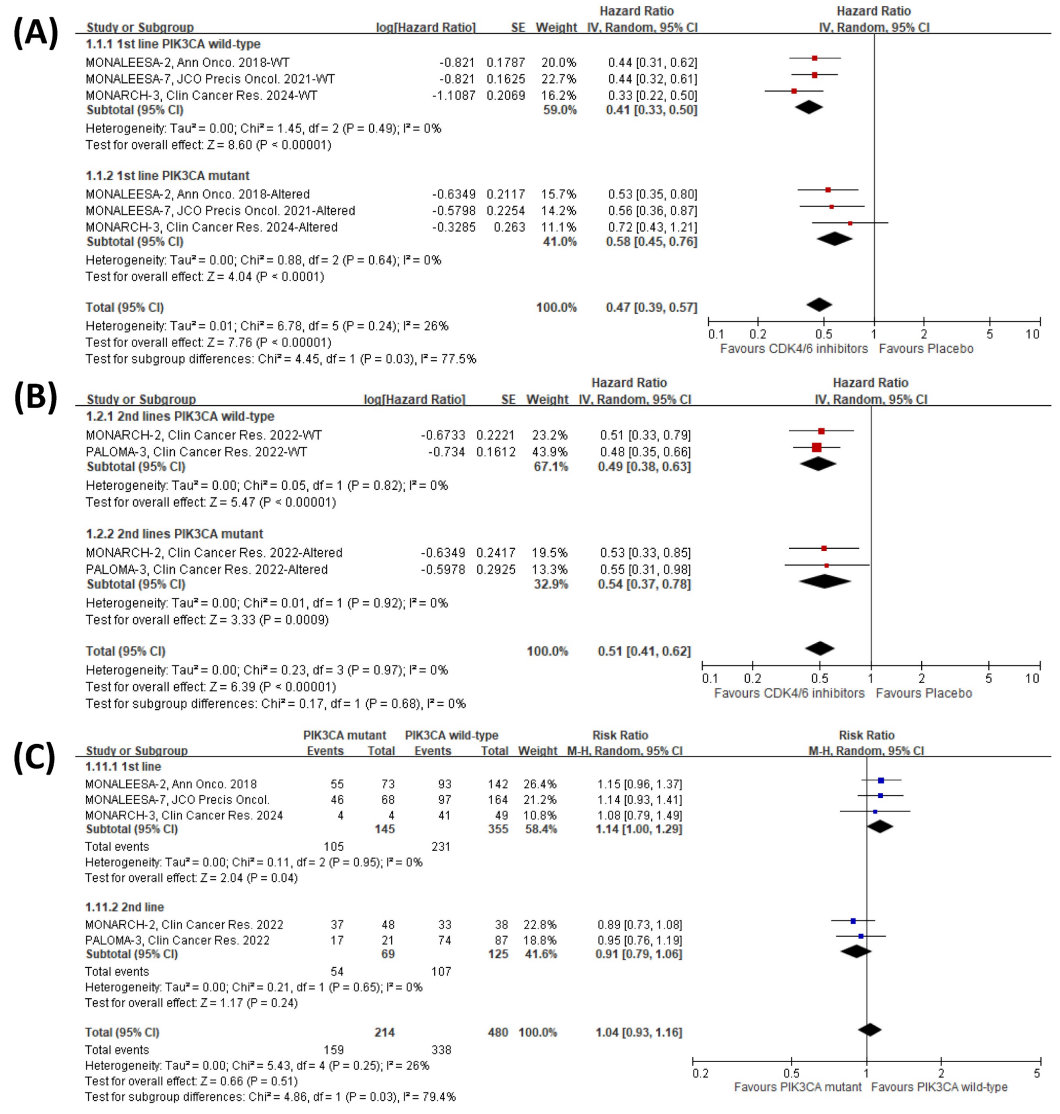
** Meta-analysis of the effects of merged lines of therapy and *PIK3CA* mutation status on PFS with CDK4/6 inhibitors.** The forest plots show hazard ratios of CDK4/6 inhibitors versus placebo for patients receiving** (A)** first-line therapy and **(B)** second or subsequent lines of therapy. **(C)** Forest plots display PFS risk ratios for *PIK3CA* mutant versus wild-type patients within the placebo control arm (endocrine therapy alone).

Our study further investigated the potential impact of *PIK3CA* status on the PFS clinical benefit of CDK4/6 inhibitors in different lines of therapy. We conducted a subgroup analysis using the individual genomic reports 37, 41. Consistently, we revealed a notable variation (*p* = 0.03) between different *PIK3CA* status patients who had not undergone previous endocrine therapy for advanced disease (first-line treatment) (Fig. 4A). Nevertheless, we noticed that the influence of *PIK3CA* status on the PFS of CDK4/6 inhibitors was nullified in patients who underwent second-line or subsequent treatments (*p* = 0.68) (Fig. 4B). Additionally, we further investigate the interaction of *PIK3CA* status in the patients within the endocrine therapy-control arm and found that *PIK3CA* status might slightly affect the PFS clinical benefit of the endocrine therapy in the patients receiving first-line treatment; however, the influence was not significant in patients who received prior therapy (Fig. 4C). These results suggest that *PIK3CA* status may significantly impact the clinical benefit of CDK4/6 inhibitors on PFS, especially in patients without prior treatment.

### Meta-analysis for the effects of CDK4/6 inhibitors on OS

**Figure 5:**
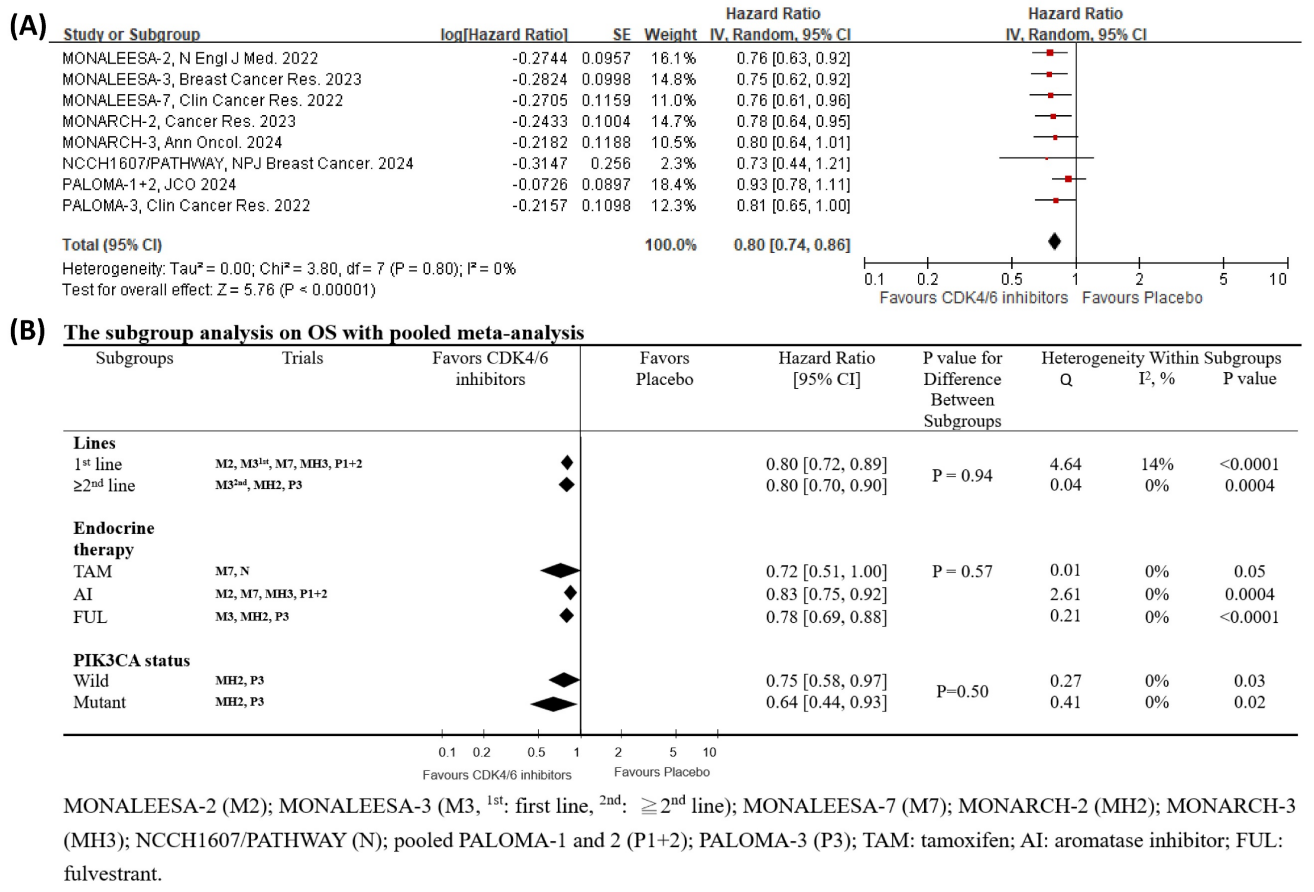
**Pooled meta-analysis and subgroup analysis of the effects of CDK4/6 inhibitors on OS. (A)** The forest plot shows the hazard ratios of CDK4/6 inhibitors versus placebo on OS for the included clinical trials.** (B)** Subgroup analysis compares the hazard ratios of CDK4/6 inhibitors versus placebo on OS across different subgroups.

Drawing from the latest OS reports 12, 18, 19, 31, 42-45, we analyzed 9 RCTs that specifically examined the impact of CDK4/6 inhibitors on OS. The outcomes were encouraging, indicating a significant reduction in HR for CDK4/6 inhibitors, as depicted in Fig. 5A [HR 0.80 (95% CI 0.74-0.86), *p* < 0.00001]. The heterogeneity test for this outcome was insignificant (I^2^ = 0 %, tau^2^ = 0, Q =3.80, *p* = 0.80), and the post-hoc power test confirmed a remarkable 100% effectiveness.

We further conducted a subgroup analysis to examine the interactions of *PIK3CA* status, different lines of therapy, and the category of combined endocrine therapy on the OS effectiveness of CDK4/6 inhibitors 13, 46. The interaction findings from the subgroup analysis, presented in Fig. 5B, were insignificant (lines of therapy, *p* = 0.94; types of endocrine therapy, *p* = 0.57; *PIK3CA* status interaction, *p* = 0.50). The subgroup analyses demonstrated a clinical benefit of CDK4/6 inhibitors except for the combination with tamoxifen (HR 0.72, *p* = 0.05, Fig. 5B). Additionally, the meta-regression consistently revealed that none of the potential factors had a statistically significant impact on OS (Table 1).

### The sensitivity analysis for clinical benefits of CDK4/6 inhibitors in the pooled meta-analysis

In our pooled meta-analysis, we conducted a sensitivity analysis to evaluate the clinical benefits of CDK4/6 inhibitors in terms of OS and PFS. This process excluded RCTs categorized as open-label, such as PALOMA-1 RCT. The clinical benefits of CDK4/6 inhibitors remained significant in terms of PFS (HR 0.56, 95% CI 0.52-0.59, *p* < 0.00001) and OS (HR 0.80, 95% CI 0.74-0.86, *p* < 0.00001) as shown in Supplementary Fig. 2. The heterogeneity test for clinical benefits outcome was still insignificant for both PFS (I^2^ = 0 %, tau^2^ = 0, Q = 12.20, *p* = 0.51) and OS (I^2^ = 0 %, tau^2^ = 0, Q = 3.94, *p* = 0.79).

### Network meta-analysis for the clinical outcomes of CDK4/6 inhibitors in HR+ and HER2- advanced breast cancer patients

**Figure 6:**
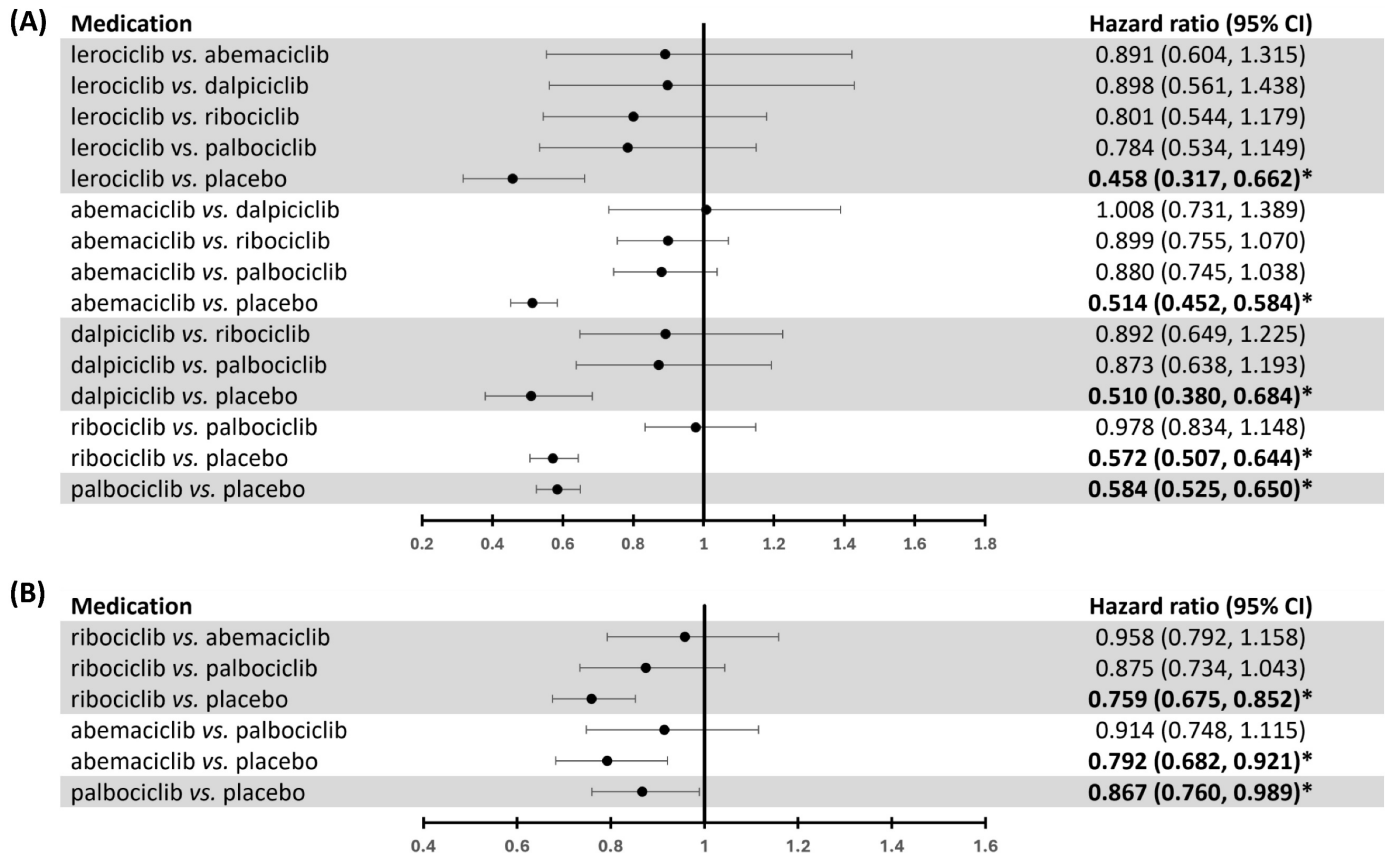
** Network meta-analysis of the effects of CDK4/6 inhibitors on PFS and OS.** This network meta-analysis compares the effects of individual CDK4/6 inhibitors on **(A)** PFS and **(B)** OS, including hazard ratio (HR) and 95% confidence interval (CI). * indicates *p* < 0.05.

Our study aimed to compare the clinical benefits of CDK4/6 inhibitors and highlight the significance of selecting the best option using network meta-analysis (Supplementary Figs. 3-5 show the diagram of network meta-analysis). There were no significant differences in PFS between CDK4/6 inhibitors (Fig. 6A). Clinical benefits of individual CDK4/6 inhibitors in comparison to placebo on PFS were observed (Fig. 6A). For ranking the probability of PFS, lerociclib was identified as having the highest clinical benefit (lerociclib>abemaciclib>dalpiciclib>ribociclib>palbociclib, Supplementary Table 3). The heterogeneity was insignificant (*p* = 0.5809).

We further dissect the effects of the lines of therapy and the interaction of *PI3K* status on PFS using individual network meta-analysis. There were no significant differences in PFS between CDK4/6 inhibitors in first-line and second- or subsequent-line therapy patients (Supplementary Fig. 6). (Ranking probability of PFS in first-line therapy: dalpiciclib > abemaciclib > ribociclib > palbociclib; in second line or beyond therapy: lerociclib > abemaciclib > palbociclib > ribociclib, Supplementary Table 4). The heterogeneity was insignificant (first-line therapy: *p* = 0.7290; second line or beyond therapy: *p* = 0.1952).

Consistently, we also found no significant differences in PFS between different CDK4/6 inhibitors in different *PIK3CA* statuses (Supplementary Fig. 7). Our data indicated that abemaciclib might deliver the highest PFS benefit in *PIK3CA* wild-type patients (abemaciclib>palbociclib>ribociclib, Supplementary Table 5), while palbociclib yield the most PFS benefit among *PIK3CA* mutant patients (palbociclib > abemaciclib > ribociclib, Supplementary Table 5). The heterogeneity was insignificant (*PIK3CA* wild-type: *p* = 0.1515; *PIK3CA* mutant: *p* = 0.3910).

Our investigation demonstrated no significant differences in OS among the different CDK4/6 inhibitors (Fig. 6B). Nevertheless, each CDK4/6 inhibitor displayed a clinical OS benefit (Fig. 6B). We further conducted an individual network meta-analysis to analyze the effects of lines of therapy on OS. We found that ribociclib had a significant OS benefit compared to palbociclib in the first-line setting (HR 0.799, 95% CI 0.642-0.993) (Supplementary Fig. 8). In contrast, the effect was insignificant in patients who have received prior endocrine therapy (Supplementary Fig. 8).

In ranking probability, ribociclib might have the highest OS benefit (ribociclib>abemaciclib>palbociclib, Supplementary Table 6). The heterogeneity was insignificant (*p* = 0.9088). Regarding OS in the first-line therapy, ribociclib exhibited the highest likelihood of providing a significant clinical benefit (ribociclib>abemaciclib>palbociclib, Supplementary Table 7). In the second or later line in therapy, abemaciclib was identified as the treatment most likely to yield the highest clinical benefit for OS (abemaciclib>ribociclib>palbociclib, Supplementary Table 7). The heterogeneity was insignificant (first-line therapy: *p* = 0.7564; second line or beyond therapy: NA).

## Discussion

The present study confirms that CDK4/6 inhibitors provide clinical benefits, including OS benefit consistent with PFS, to HR+/HER2- advanced breast cancer patients (Figs. 2 and 5A). Our findings indicate that the clinical benefits of CDK4/6 inhibitors are consistent across different lines of therapy, categories of combined endocrine therapy, follow-up duration, age, ethnicity, ECOG performance status, previous chemotherapy or endocrine therapy, and metastatic status (Table 1, Figs. 3 and 5B). Notably, we discovered that the *PIK3CA* status might influence the efficacy of CDK4/6 inhibitors in terms of PFS for the first line of therapy (Figs. 3-4).

Up to 40% of HR+ metastatic breast cancer patients may harbor *PIK3CA* mutations, and the PI3K/AKT/mammalian target of rapamycin pathway is implicated as a common escape route for endocrine therapy 9. Active PI3K signaling (due to *PIK3CA* mutation) may interact significantly with the ER signaling pathway. Consequently, the negative prognostic impact of *PIK3CA* mutation is more pronounced in the first-line setting, where tumors are relatively naïve to endocrine therapy and CDK4/6 inhibitors. In contrast, the mechanisms of endocrine resistance are far more complex in the second line or beyond 47, potentially diluting the biological impact of *PIK3CA* mutation on CDK4/6 inhibitor combinations. Indeed, upon analyzing the interaction of *PIK3CA* status in patients undergoing only endocrine therapy (control arms of the CDK4/6 inhibitor clinical trials), it was observed that *PIK3CA* mutations may negatively impact the PFS clinical benefit for those receiving first-line treatment. However, this effect was not significant in patients who had previously undergone therapy (Fig. 4C). Moreover, an exploratory biomarker analysis from the postMONARCH trial—which evaluated abemaciclib with a modified endocrine therapy backbone after disease recurrence on prior CDK4/6 inhibitors and endocrine therapy—demonstrated consistent clinical benefits across genomic subgroups, including *PIK3CA* or *ESR1* status, supporting the diminished impact of *PIK3CA* status in later-line settings 48. In the first-line setting, combining inavolisib (a PI3K inhibitor) with palbociclib and fulvestrant recently demonstrated a clinically meaningful improvement in PFS in patients with *PIK3CA*-mutated, HR+, HER2-, endocrine-resistant, locally advanced or metastatic breast cancer 49. However, it should be noted that there was a higher incidence of Grade 3/4 adverse events, including thrombocytopenia (14.2% vs. 4.3%), stomatitis or mucosal inflammation (5.6% vs. 0%), hyperglycemia (5.6% vs. 0%), and diarrhea (3.7% vs. 0%) 49. Our findings have important implications for personalized treatment strategies in naïve-specific populations.

Despite the promising therapeutic efficacy of CDK4/6 inhibitors in breast cancer, intrinsic (de novo) and acquired resistance remain significant challenges to effective disease management 50, 51. A systematic review of biomarkers predicting drug response, including intrinsic and acquired resistance to CDK4/6 inhibition in metastatic breast cancer, has been conducted 52. These include genetic alterations among key players in CDK4/6 cell cycle regulation and cross-talk pathways. Accordingly, the retinoblastoma gene 1 (*RB1*), a key tumor suppressor gene, is one of the most extensively studied factors in drug resistance to CDK4/6 inhibitors 52. Genetic mutations in the *RB1* gene or other molecular mechanisms leading to *RB1* function loss account for up to 9 % of patients who develop acquired resistance 52, 53. Additionally, abnormal cyclin E1/E2 signaling and excessive CDK2 activity have been identified as alternative resistance mechanisms, particularly in patients with prior endocrine resistance 52. Emerging evidence has also linked novel resistance mechanisms, such as FAT Atypical Cadherin 1 loss, which leads to increased CDK6 activity 52, 54. However, given the genetic heterogeneity among samples in the analyzed RCTs, differences in testing methodologies, and the fact that not all genomic alterations were assessed, other driver mutations contributing to primary resistance to CDK4/6 inhibitors and/or endocrine therapy may also influence the impact of *PIK3CA* status in combination therapy, particularly in the first-line setting. In the FLIPPER trial, which evaluated first-line treatment with palbociclib and fulvestrant, ≥1 mutation in *PIK3CA* + *TP53* was associated with early progression (≤ 12 months) regardless of the treatment arm 55. Additionally, ≥ 2 mutations in *PIK3CA* + *TP53* correlated with poorer PFS and OS outcomes 55. Notably, patients with ≥ 2 mutations in *PIK3CA* + *TP53* in the control arm exhibited early progression and worse OS, whereas this association was not observed in the palbociclib arm 55. These findings suggest that, beyond *PIK3CA* status, other primary resistance mechanisms may collectively influence the therapeutic response to CDK4/6 inhibitor combination therapy in the first-line setting. A more comprehensive, well-designed study is warranted to further elucidate these interactions.

Apart from ER, no other reliable biomarkers are currently utilized for selecting combination therapy involving CDK4/6 inhibitors and endocrine therapy in breast cancer. ER loss, which occurs in only a minority of ER-positive breast cancer patients during treatment, is linked to resistance to endocrine therapy and remains a significant predictor of the effectiveness of CDK4/6 inhibitors combined with endocrine therapy 56. In some cases, CDK4/6 inhibition has shown the potential to delay the onset of endocrine resistance, suggesting that resistance to combination therapy is primarily driven by resistance to the endocrine therapy backbone. This hypothesis may be supported by the circulating tumor DNA biomarker analysis from the MONARCH-3 trial, which found a lower incidence of* ESR1* mutations under abemaciclib intervention 57.

Although the *ESR1* mutation may play an important role in the progression of advanced HR+, HER2- breast cancer patients 58, its clinical role in CDK4/6 inhibitor resistance appears minimal 53. Subgroup analysis from the Phase I/II TRINITI-1 trial suggests that the presence of *ESR1* mutations is associated with a poorer prognosis in patients with advanced HR+/HER2- breast cancer treated with a combination of exemestane, ribociclib, and everolimus following progression on prior therapies 59. However, recent real-world analyses further suggest that *ESR1* status may not significantly affect the time-to-next-treatment of CDK4/6 inhibitor regimens and the choice of concomitant endocrine therapy for ESR1 mutations is more important than the efficacy of CDK4/6 inhibitors themselves, indicating that *ESR1* variants might not be associated with CDK4/6 inhibitor resistance 60. According to the PALOMA-3 study, *ESR1* mutations are dynamic and reflect resistance to prior aromatase inhibitor therapy; however, they may have limited utility as predictive biomarkers for the efficacy of CDK4/6 inhibitors 61, 62. Mechanistically, *ESR1* mutations may predominantly contribute to resistance against endocrine therapy 63. Indeed, the recent EMBER-3 trial demonstrated that imlunestrant, a novel oral selective estrogen receptor degrader, showed superior efficacy compared to standard endocrine therapy in endocrine therapy-pretreated/ER+/HER2- advanced breast cancer patients harboring *ESR1* mutations 64. Moreover, when combined with abemaciclib, imlunestrant significantly improved PFS compared to imlunestrant monotherapy, regardless of *ESR1* mutation status 64. A retrospective pharmacogenetic study further demonstrated that *ESR1* mutations are independent predictors of resistance to adjuvant endocrine therapy; however, no difference in PFS was observed between patients with or without *ESR1* mutations when treated with CDK4/6 inhibitors as first-line therapy 65. The PADA-1 trial was specifically designed to address this issue by monitoring *ESR1* mutation status in patients receiving combination therapy with an aromatase inhibitor and palbociclib 66. Upon detection of rising *ESR1* mutations, switching to fulvestrant plus palbociclib significantly improved PFS compared to continuing aromatase inhibitor plus palbociclib, highlighting the critical role of *ESR1* mutations in mediating resistance to endocrine therapy 66.

Other cell cycle regulators, such as mouse double minute 2 homolog-TP53, might contribute to CDK4/6 inhibitor resistance 67, making them promising targets. Although patients with wild-type *TP53* exhibited numerically longer PFS than those with *TP53* alterations in the MONALEESA-2 study 37, the interaction between *TP53* status and PFS was not statistically significant across the MONALEESA Phase III trials 9. Despite a significant enrichment of *TP53* mutations in tumor samples resistant to CDK4/6 inhibitors, *in vitro* studies have demonstrated comparable sensitivity to CDK4/6 inhibitors regardless of *TP53* status 7. However, a recent genomic cohort study demonstrated that* TP53* mutations are associated with a lack of long-term disease control in patients with metastatic HR+/HER2- breast cancer treated with first-line CDK4/6 inhibitors and endocrine therapy 68. Additionally, the study revealed that *TP53* loss promotes CDK2 activation, thereby facilitating cell-cycle re-entry and tumor progression in an *in vitro* breast cancer model 68. Moreover, *TP53* mutations may contribute to primary resistance to endocrine therapy 69. Given that *TP53* might contribute to the reduced efficacy of both CDK4/6 inhibitors and endocrine therapy, as well as their combination, it might be challenging to discern a distinct impact of *TP53* mutations on the HR of CDK4/6 inhibitor combination arms compared to those of endocrine therapy alone. We further conducted an exploratory analysis to evaluate the impact of *TP53* and *ESR1* mutations on PFS events within the CDK4/6 inhibitor plus endocrine therapy group and the endocrine therapy alone group, respectively. Our findings tentatively suggest that patients with *TP53* wild-type status might experience a favorable PFS outcome, regardless of whether they received CDK4/6 inhibitor plus endocrine therapy or placebo plus endocrine therapy (Supplementary Figs. 9A and B). In contrast, *ESR1* mutation status was not significantly associated with PFS outcomes (Supplementary Figs. 9C and D). However, these results should be interpreted carefully, as they were derived without multivariate analysis or randomization and may be influenced by potential confounding factors. Furthermore, the *ESR1* mutations are acquired over time and tend to emerge with increasing frequency during the course of endocrine therapy, indicating that an initially wild-type *ESR1* status may evolve during treatment 70, 71. Given the uncertainties surrounding current clinical trials investigating the genomic impact on CDK4/6 inhibitor efficacy, further rigorous clinical-genomic studies with meticulous methods are warranted to enhance the translational relevance of these findings.

The updated genomic report of MONALEESA trials recently identified *PI3KC*A (40.6%) as the most prevalent baseline gene alteration, followed by *TP53* (28.5%) 72. Although *RB1* alterations were relatively rare at baseline among patients treated with ribociclib (1.6%), their prevalence increased to 10% at the end-of-treatment (EOT) when comparing genomic alterations in paired baseline and EOT circulating tumor DNA samples from the ribociclib arm 72. Additionally, *RB1* gene alterations might interact with the PFS benefit of ribociclib in the MONALEESA trials 9. Similar findings were reported in the PALOMA-3 trial, where *RB1* mutation prevalence increased to 4.7% at EOT (predominantly in the palbociclib plus fulvestrant arm) 53, and the *RB1* loss or loss of heterozygosity was associated with worse prognosis in this trial 73. Moreover, the MONARCH-3 trial demonstrated a higher frequency of acquired *RB1* alterations in the abemaciclib treatment arm compared to the placebo (5% *vs.* 0%) 14. However, no significant interaction effect was observed between *RB1* mRNA gene expression and treatment outcomes in the PALOMA-2 56 and PALOMA-3 trials 74.

Acquired cyclin E1 (*CCNE1*) amplification has been identified in palbociclib- and abemaciclib-resistant preclinical models 75. Although high *CCNE1* mRNA expression 74 and *CCNE1* copy number gain 73 were associated with poorer PFS, no patients exhibited acquired *CCNE1* amplification at EOT in the PALOMA-3 trial 53. Additionally, *CCNE1* RNA expression did not show a significant interaction with the PFS benefit of CDK4/6 inhibitors in the PALOMA-2 trial 56. Furthermore, alterations in cell cycle-related genes, including cyclin D1 (*CCND1*), *CCND2*, *CDK4*, *CDK5*, cyclin dependent kinase inhibitor 2A, *CCNE1*, *RB1*, and *TP53*, did not significantly influence PFS benefit in the MONARCH-3 trial 14. *CCND1* genomic alterations were observed in 8.5-13% of patients in the MONARCH-3 14 and MONALEESA 9 trials. Our analysis further confirmed that *CCND1* alterations had no significant interaction with the PFS benefit of CDK4/6 inhibitors (*p* for interaction: 0.71, Supplementary Fig. 10), consistent with findings on *CCND1* RNA expression in the PALOMA-2 56 and PALOMA-3 trials 74. In the MONALEESA trials, *BRCA1/2* gene alterations were identified in 4% of patients 9, while in the MONARCH-3 trial, acquired *BRCA2* genomic alteration were more frequent in the abemaciclib arm than in the placebo (4% *vs.* 0%, *P* = 0.029) 14. However, *BRCA1/2* genomic alterations showed no significant interaction with PFS benefit in the MONALEESA trials 9. Given that these biomarkers were present in less than 10% of patients, the statistical power of these findings may be limited by sample size. Moreover, we found that *PIK3CA* alterations might not exhibit significant mutual exclusivity or co-occurrence with *TP53*, *ESR1*, *RB1*, *CCNE1*, *CCND1*, and *BRCA1/2* alterations in HR+/HER2- metastatic breast cancer patients by analysis of the established genomic dataset 76 (Supplementary Table 8). Further large-scale genomic evaluations are warranted to validate these observations.

When comparing the selectivity and potency of CDK4/6 inhibitors, it has been demonstrated that ribociclib preferentially inhibits CDK4 over CDK6. Conversely, palbociclib exhibits similar potency in targeting CDK4 and CDK6 77. Previous evidence indicated that abemaciclib displays the highest potency in inhibiting CDK4 among the current recommended CDK4/6 inhibitors for advanced breast cancer 77. Additionally, abemaciclib has demonstrated some characteristics as a pan-CDK inhibitor 77, suggesting it may have additional targets beyond CDK4/6. However, similar to the other evidence 15, our study demonstrated no statistically significant difference in clinical benefits among the CDK4/6 inhibitors. Although our analysis suggests that ribociclib may exhibit greater clinical efficacy than palbociclib in terms of OS for first-line treatment (Supplementary Fig. 8) and reveals the ranking probability of CDK4/6 inhibitors in specific clinical outcomes of individual populations (Supplementary Tables 3-7), the limited evidence underscores the need for additional research to determine the most efficacious treatment option for clinical settings more conclusively. Moreover, it is worth noting that the effectiveness of lerociclib and dalpiciclib can only be demonstrated in the local region RCT. Further clinical trials and real-world analyses are imperative to address this knowledge gap.

CDK4/6 inhibitors consistently enhance PFS and OS in HR+/HER2- advanced breast cancer patients, regardless of treatment line, endocrine therapy type, *ESR1* and *TP53* status, follow-up duration, age, ethnicity, ECOG performance status, previous chemotherapy or endocrine therapy, and metastatic status. *PIK3CA* status may impact the PFS of CDK4/6 inhibitors in patients who are endocrine therapy-naïve for advanced disease, but not in those previously treated. While clinical benefits among CDK4/6 inhibitors are largely comparable, limited evidence suggests ribociclib may offer superior OS benefit over palbociclib in first-line clinical settings, with ranking probabilities varying by clinical context. These findings highlight the need to consider *PI3KCA* status when selecting first-line CDK4/6 inhibitors for HR+/HER2- advanced breast cancer. The efficacy of CDK4/6 inhibitors may differ based on the specific CDK4/6 inhibitor and clinical scenario. Further research and prospective validation in dedicated studies are essential to comprehensively assess the impact of *PIK3CA* status and different CDK4/6 inhibitors on clinical outcomes in HR+/HER2- advanced breast cancer.

## Supplementary Material

Supplementary figures and tables.

Supplementary data.

## Figures and Tables

**Table 1:** Meta-regression analysis for potential factors in clinical benefit of CDK4/6 inhibitors on PFS and OS.

PFS	Coefficient	SE	95%	Z	2-sided P-value
*Median follow-up (months)*	0.0009	0.0016	-0.0023-0.0040	0.54	0.5910
*Median age (yrs)*	0.0023	0.0061	-0.0097-0.0143	0.38	0.7063
*Race (White, %)*	0.0026	0.0041	-0.0054-0.0106	0.63	0.5286
*ECOG = 0 (%)*	-0.0007	0.0044	-0.0094-0.0080	-0.16	0.8751
*Previous C/T (%)*	-0.0001	0.0027	-0.0055-0.0053	-0.03	0.9767
*Previous ET (%)*	-0.0003	0.0020	-0.0042-0.0036	-0.16	0.8714
*Metastatic sites ≥3 (%)*	0.0054	0.0073	-0.0088-0.0197	0.75	0.4542
*Visceral metastases (%)*	-0.0025	0.0076	-0.0174-0.0125	-0.32	0.7471
***OS***	**Coefficient**	**SE**	**95%**	**Z**	**2-sided** **P-value**
*Median follow-up (months)*	0.0028	0.0029	-0.0029-0.0086	0.96	0.3355
*Median age (yrs)*	0.0032	0.0067	-0.0100-0.0163	0.47	0.6369
*Race (White, %)*	0.0000	0.0041	-0.0080-0.0081	0.01	0.9908
*ECOG = 0 (%)*	-0.0101	0.0068	-0.0234-0.0031	-1.50	0.1344
*Previous C/T (%)*	-0.0029	0.0053	-0.0134-0.0075	-0.55	0.5818
*Previous ET (%)*	-0.0011	0.0031	-0.0072-0.0051	-0.34	0.7335
*Metastatic sites ≥3 (%)*	0.0128	0.0097	-0.0062-0.0318	1.32	0.1859
*Visceral metastases (%)*	-0.0149	0.0091	-0.0328-0.0030	-1.63	0.1025

C/T: chemotherapy; ET: endocrine therapy

## Abbreviations

CCND1: cyclin D1
CCNE1: cyclin E1
CI: confidence interval
CDK: cyclin-dependent kinase
CDK4/6i: Cyclin-dependent kinase 4/6 inhibitors
CINeMA: Confidence in Network Meta-Analysis
EOT: end-of-treatment
ESR1: estrogen receptor 1
ER: estrogen receptor
HR: hazard ratio
HR+: hormone receptor-positive
HER2: human epidermal growth factor receptor-2
HER2-: human epidermal growth factor receptor-2 negative
OS: overall survival
PI3K: phosphatidylinositol 3-kinase
PIK3CA: phosphatidylinositol-4,5-bisphosphate 3-kinase catalytic subunit alpha
PFS: progression-free survival
PRISMA: Preferred reporting of systematic reviews and meta-analyses
RB1: Retinoblastoma gene 1
RCT: randomized control trial
RR: risk ratio
TP53: tumor protein p53
