# Supplementary material for: Impact of PIK3CA Mutations on the Clinical Benefits of CDK 4/6 Inhibitors in HR+/HER2- Advanced Breast Cancer: An Updated Pairwise and Network Meta-Analysis
Source: J Cancer. 2025 Jul 4;16(10):3065–79. doi: 10.7150/jca.111362 (PMC12305603; doi:10.7150/jca.111362)
Supplement: Supplementary file 1 — Supplementary figures and tables. [file jcav16p3065s1.pdf]

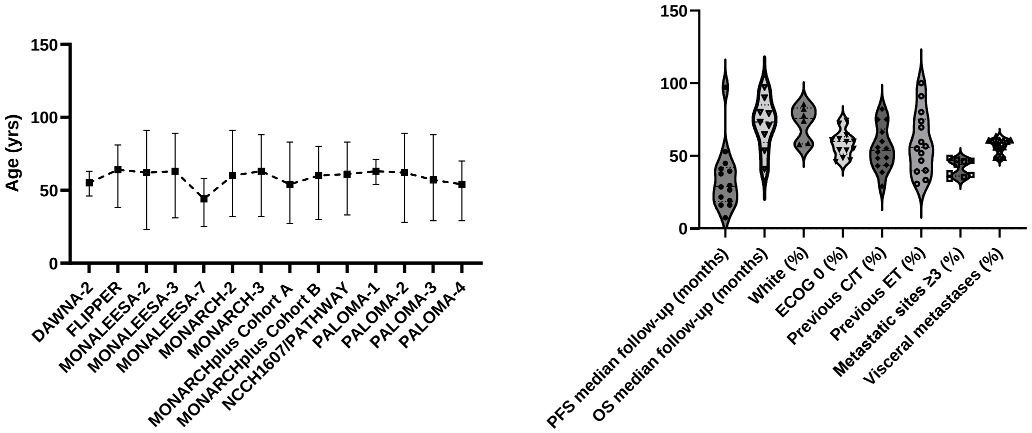

Supplementary Fig. 1. Baseline patient characteristics similarity across randomized controlled trials. Most comparisons revealed similar patient characteristics, with a few exceptions showing relatively low or high levels. The meta-regressions, which considered various demographic and clinical characteristics such as median age, follow-up time, percentage of the white ethnic group, ECOG score = 0, previous chemotherapy, previous endocrine therapy, metastatic sites ≥ 3, and presence of visceral metastases, indicated that these factors did not impact the estimates shown in Table 1. This reinforces the reliability of our data.

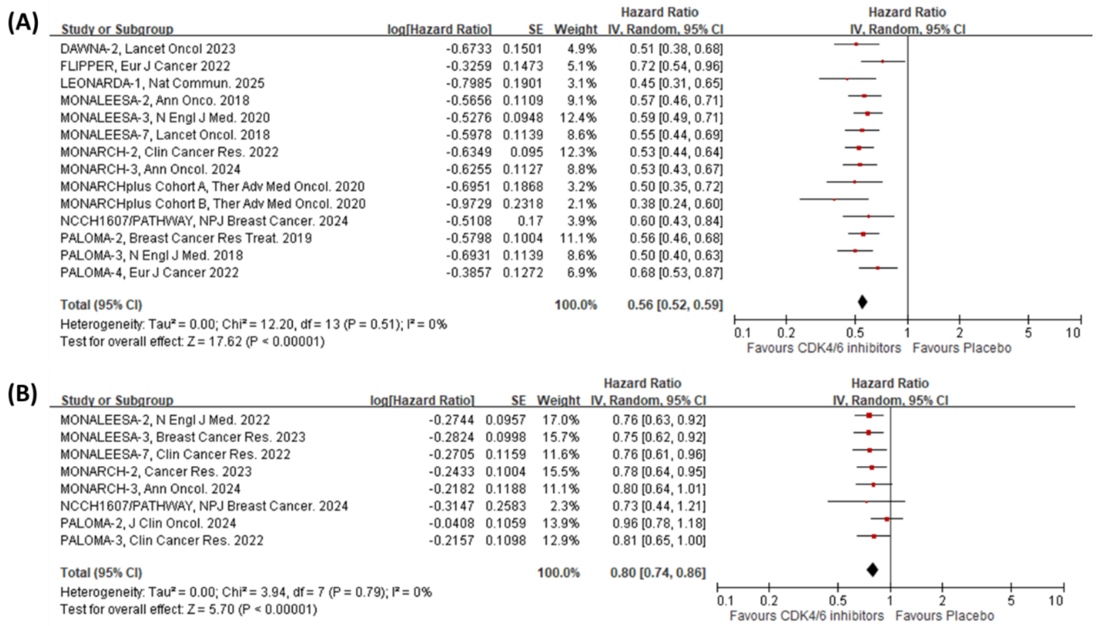

Supplementary Fig. 2 Sensitivity analysis of CDK4/6 inhibitors' effects on clinical benefits. The sensitivity analysis of the effects of CDK4/6 inhibitors on clinical benefits was performed using pooled meta-analysis. The forest plots for the sensitivity analysis of HR of CDK4/6 inhibitors versus placebo on **(A)** PFS and **(B)** OS are shown in Supplementary Fig. 2.

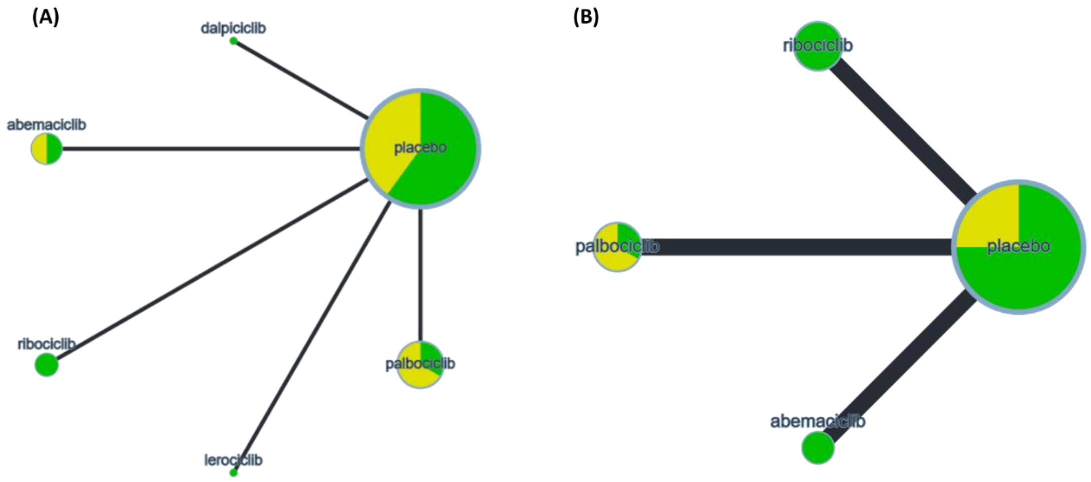

Supplementary Fig. 3. Network plots in PFS and OS analysis. The network plots for the **(A)** PFS) and **(B)** OS analyses are shown in Supplementary Fig. 3. These plots display node size proportional to the number of studies, node color indicating risk of bias (Green: low; Yellow: some concerns), and edge width by equal size.

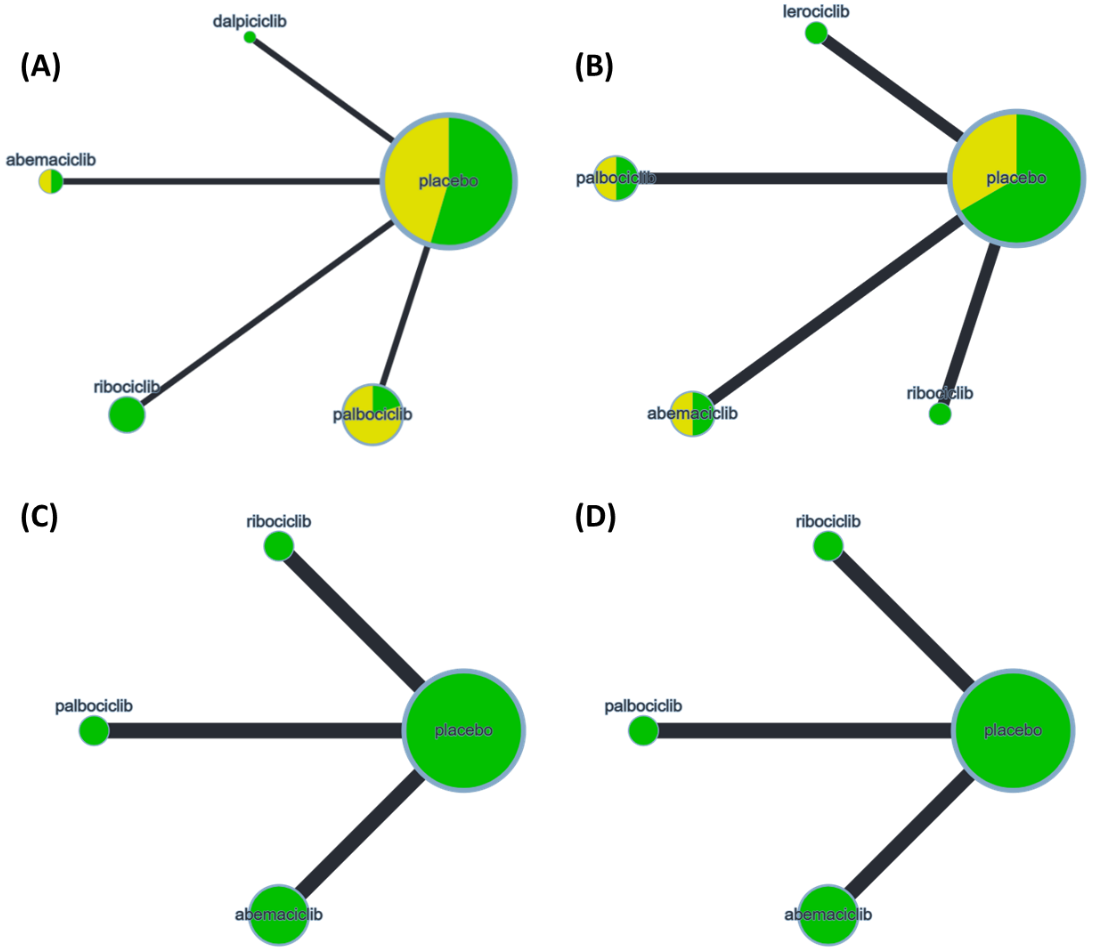

Supplementary Fig. 4. Network plots in subgroup analysis of PFS. The network plots for the subgroup analysis of PFS in patients with **(A)** 1st line therapy, **(B)** ≥ 2 lines of therapy, **(C)** *PIK3CA* wild-type, and **(D)** *PIK3CA* mutant are presented in Supplementary Fig. 4.

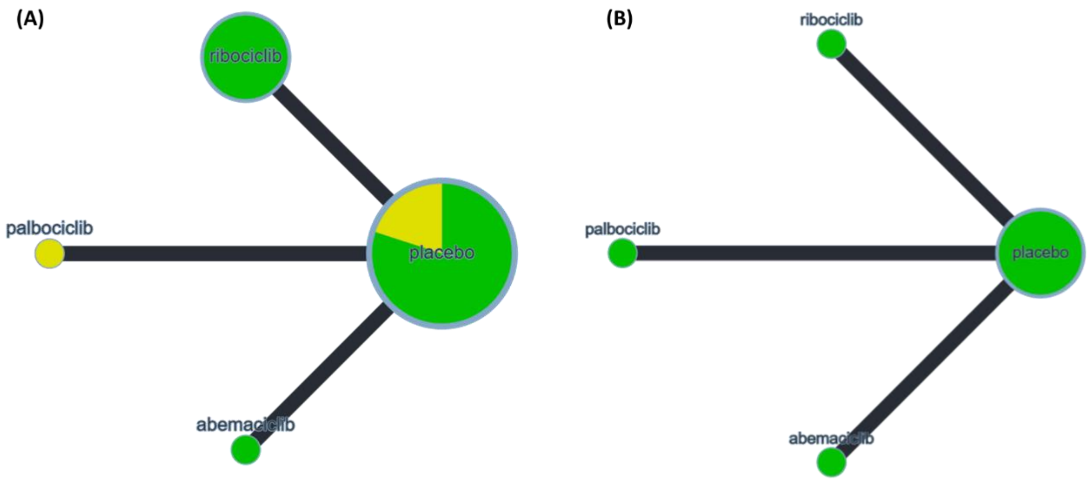

Supplementary Fig. 5. Network plots in subgroup analysis of OS. The network plots for the subgroup analysis of OS in patients receiving **(A)** 1st line therapy and **(B)** ≥ 2 lines of therapy are presented in Supplementary Fig. 5.

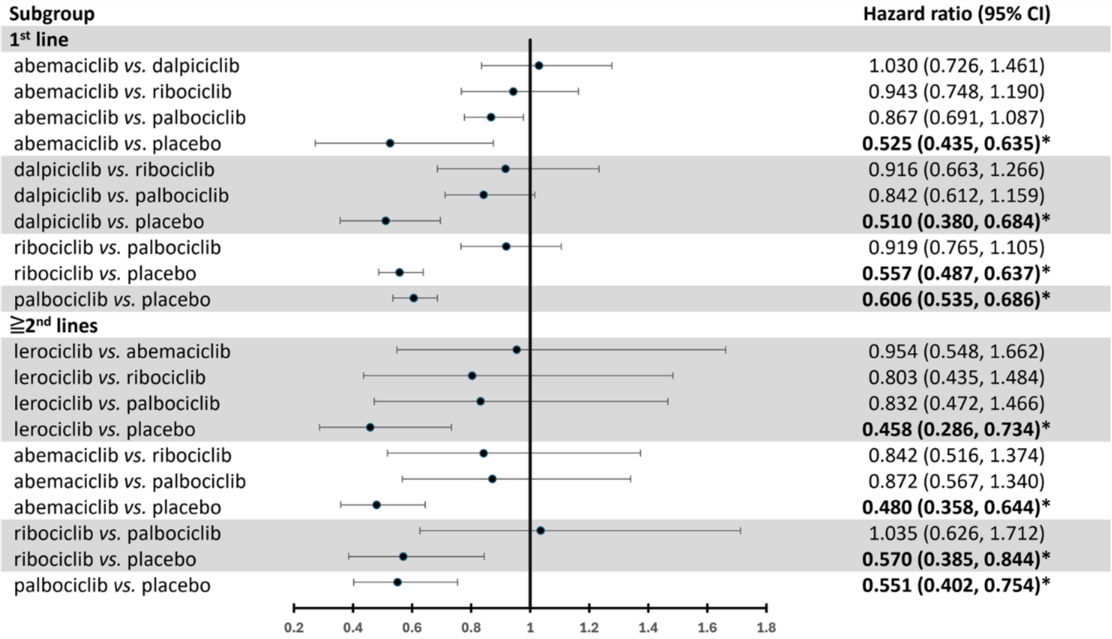

Supplementary Fig. 6. Network meta-analysis for the effects of CDK4/6 inhibitors on PFS in patients receiving individual lines of therapy. The network meta-analysis evaluates the impact of CDK4/6 inhibitors on PFS across different therapy lines. This analysis includes hazard ratio (HR) and 95% confidence interval (CI) comparisons for all strategies. *: $p < 0.05$ indicates statistical significance.

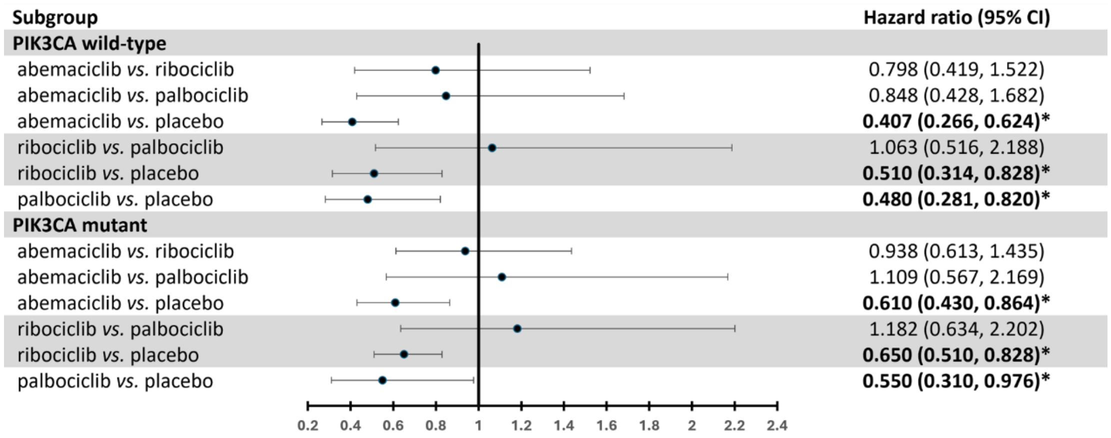

Supplementary Fig. 7. Network meta-analysis for the effects of CDK4/6 inhibitors on PFS in *PIK3CA* status subgroups. The network meta-analysis evaluates the effects of CDK4/6 inhibitors on PFS across different *PIK3CA* status subgroups. This analysis compares the effects of all strategies, including hazard ratio (HR) and 95% confidence interval (CI). *: $p < 0.05$ indicates statistical significance.

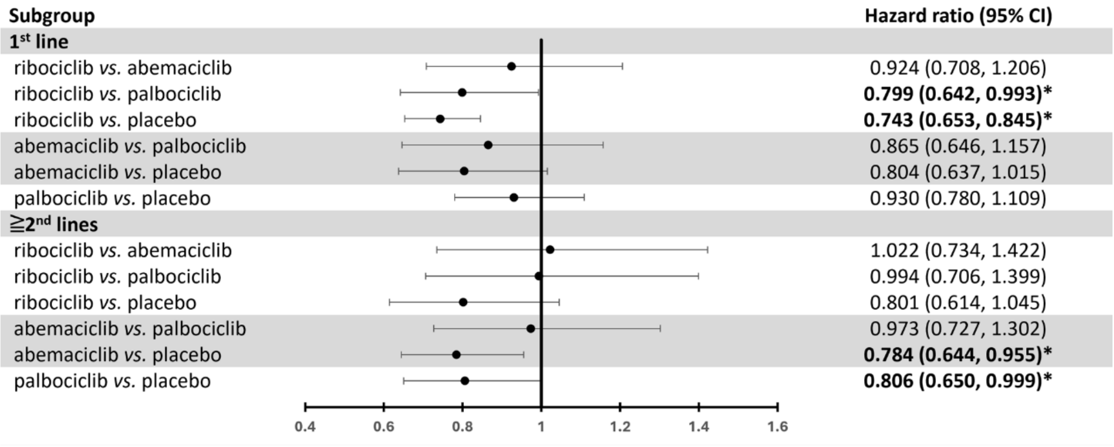

Supplementary Fig. 8. Network meta-analysis for the effects of CDK4/6 inhibitors on OS in patients with individual lines of therapy. The network meta-analysis evaluates the effects of CDK4/6 inhibitors on OS across individual lines of therapy. This analysis compares the effects of all strategies, including hazard ratio (HR) and 95% confidence interval (CI). *: $p < 0.05$ indicates statistical significance.

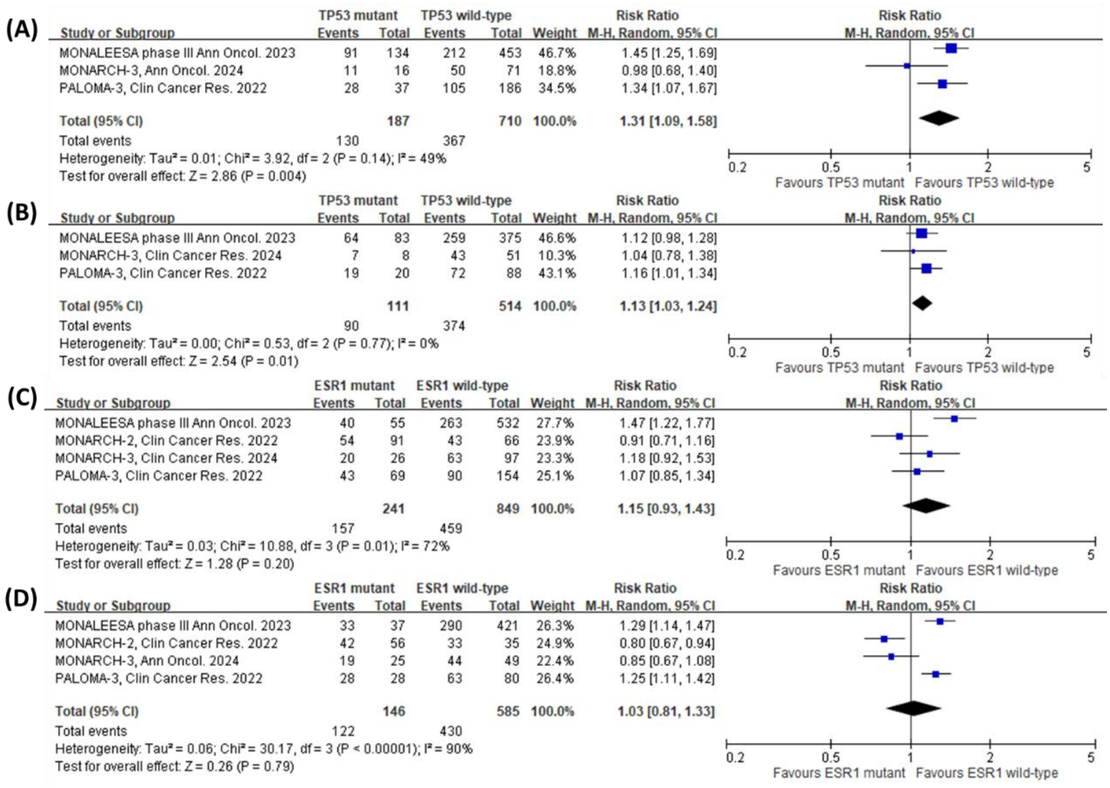

Supplementary Fig. 9. Meta-analysis of the impact of *TP53* and *ESR1* mutation status on PFS events in CDK4/6 inhibitor trials. (A-D) Forest plots illustrate the risk ratios for PFS events comparing **(A, B)** *TP53*-mutant *vs.* wild-type and **(C, D)** *ESR1*-mutant *vs.* wild-type patients within the **(A, C)** CDK4/6 inhibitor treatment arms and the **(B, D)** placebo (endocrine therapy alone) control arms.

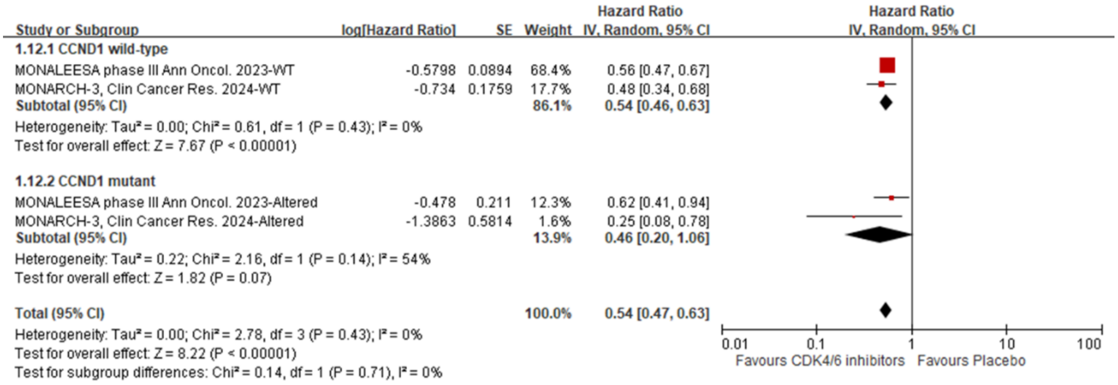

Supplementary Fig. 10. Meta-analysis of the effect of *CCND1* status on PFS with CDK4/6 inhibitors. The forest plots show hazard ratios of CDK4/6 inhibitors versus placebo across different *CCND1* status subgroups.

Supplementary Table 1. Checklist items to include when reporting a systematic review involving a network meta-analysis.

Section/Topic	Item # *	Reported on Page #
**TITLE**		
Title	**1**	1
**ABSTRACT**		
Structured summary	**2**	3
**INTRODUCTION**		
Rationale	**3**	4, 5
Objectives	**4**	5
**METHODS**		
Protocol and registration	**5**	5, 6
Eligibility criteria	**6**	5, 6
Information sources	**7**	5
Search	**8**	5
Study selection	**6**	5, 6
Data collection process	**10**	6
Data items	**11**	6
Geometry of the network	**S1**	7
Risk of bias within individual studies	**12**	6
Summary measures	**13**	6, 7
Planned methods of analysis	**14**	6, 7
Assessment of inconsistency	**S2**	7
Risk of bias across studies	**15**	6
Additional analyses	**16**	6, 7
**RESULTS**		
Study selection	**17**	7, 8
Presentation of network structure	**S3**	10
Summary of network geometry	**S4**	10
Study characteristics	**18**	8
Risk of bias within studies	**19**	8-10 (Fig. 2 and Supplementary Figs. 3-5)

Results of individual studies	**20**	8-10
Synthesis of results	**21**	8-10
Exploration for inconsistency	**S5**	7
Risk of bias across studies	**22**	8-10 (Fig. 2 and Supplementary Figs. 3-5)
Results of additional analyses	**23**	8-11
**DISCUSSION**		
Summary of evidence	**24**	11, 12
Limitations	**25**	16
Conclusions	**26**	16, 17
**FUNDING**		
Funding	**27**	17, 18

Supplementary Table 2. Baseline information of included trials (adapted from ClinicalTrials.gov)

Trial	Study Design	Study Population	Location Countries	Treatment Arms	Outcomes
**DAWNA-2 NCT03966898**	Study Phase: Phase 3<br>Allocation: Randomized<br>Masking: Double (participant, investigator)	Patients who have HR-positive and HER-2-negative recurrent/metastatic breast cancer and have not received systemic anticancer therapy	China	Dalpiciclib combination with Letrozole or Anastrozole *vs.* placebo in combination with Letrozole or Anastrozole	PFS, OS, ORR, DOR, CBR, SAE, AE
**FLIPPER NCT02690480**	Study Phase: Phase 2<br>Allocation: Randomized<br>Masking: Triple (participant, care provider, investigator)	Postmenopausal women with HR-positive/HER-2-negative metastatic breast cancer who have received ≥5 years of endocrine therapy in the adjuvant setting as a treatment for early disease and remained	Ireland, Spain	Fulvestrant in combination with palbociclib *vs.* fulvestrant plus placebo	PFS, ORR, CBR, OS, 1-year and 2-year survival probabilities, AE, patient-reported outcomes of health-related quality of life based on EORTC QLQ-C30 Global Health Status/Quality of Life and Physical Function and EORTC QLQ-BR23 Breast Module

		disease-free for > 12 months following its completion or have "de novo" metastatic diseased			
**LEONARDA-1 NCT05054751**	Study Phase: Phase 3<br>Allocation: Randomized<br>Masking: Quadruple (participant, care provider, investigator, outcomes assessor)	HR+, HER2- locally advanced or metastatic breast cancer who have progressed on prior endocrine therapy	China	Lerociclib combined with fulvestrant vs. placebo with fulvestrant	PFS, OS, ORR, DOR, DCR, CBR, SAE, AE, TEAE, Pharmacokinetics (Cmax, Tmax, AUC)
**MONALEESA-2 NCT01958021**	Study Phase: Phase 3<br>Allocation: Randomized<br>Masking: Quadruple (Participant, care provider, investigator, outcomes assessor)	Postmenopausal women with HR-positive, HER2-negative advanced breast cancer who received no prior treatment for advanced disease	Argentina, Australia, Austria, Belgium, Brazil, Canada, Czechia, Denmark, Finland, France, Germany, Hungary, Ireland, Israel, Italy, Republic of Korea, Lebanon,	Ribociclib in combination with letrozole *vs.* placebo with letrzole	PFS, ORR, OS, CBR, time to definitive deterioration of ECOG Performance Status in one category of the score, safety and tolerability, time to definitive 10% deterioration in the Global Health Status/Quality of Life (QOL) Scale Score (EORTC QLQ-C30),

			Netherlands, Norway, Russian Federation, Singapore, South Africa, Spain, Sweden, Taiwan, Thailand, Turkey, United Kingdom, United States		QTc interval
**MONALEESA-3 NCT02422615**	Study Phase: Phase 3<br>Allocation: Randomized<br>Masking: Quadruple (participant, care provider, investigator, outcomes assessor)	Men and postmenopausal women diagnosed with HR+, HER2-negative advanced breast cancer who have received no or only one line of prior endocrine treatment.	Australia, Austria, Belgium, Bulgaria, Canada, Colombia, Czechia, Denmark, France, Germany, Hungary, Italy, Jordan, Republic of Korea, Lebanon, Malaysia, Mexico, Netherlands, Norway, Poland, Portugal, Russian Federation, Singapore, Spain, Sweden, Switzerland, Thailand, Turkey,	Fulvestrant in combination with ribociclib *vs.* fulvestrant with placebo	PFS, OS, ORR, CBR, TTR, DOR, time to definitive deterioration of ECOG Performance Status in one score category, time to definitive 10% deterioration in the Global Health Status/Quality of Life (GHS/QoL) Scale Score of the European Organization for Research and Treatment of Cancer's Core Quality of Life Questionnaire, change from baseline in the GHS/QoL Scale Score of the EORTC QLQ-C30, ribociclib/LEQ803 plasma

			United Kingdom, United States		concentrations)
**MONALEESA-7 NCT02278120**	Study Phase: Phase 3 Allocation: Randomized Masking: Quadruple (participant, care provider, investigator, outcomes assessor)	Premenopausal women with HR+, HER2- advanced breast cancer. Exclusion: patients who had received any prior hormonal anti-cancer therapy for advanced breast cancer, except for ≤ 14 days of tamoxifen or NSAI ± goserelin for advanced breast cancer prior to randomization.	Argentina, Australia, Belgium, Brazil, Bulgaria, Canada, Colombia, France, Germany, Greece, Hong Kong, Hungary, India, Italy, Republic of Korea, Lebanon, Malaysia, Mexico, Poland, Portugal, Russian Federation, Saudi Arabia, Singapore, Spain, Switzerland, Taiwan, Thailand, Turkey, United Arab Emirates, United States	Ribociclib + goserelin + tamoxifen or a NSAI (letrozole or anastrozole) *vs.* placebo + goserelin + tamoxifen or a NSAI	PFS, OS, ORR, CBR, TTR, DOR, time to definitive deterioration of Eastern Cooperative Oncology Group Performance Status (ECOG PS) by at least one category of the score, time to definitive 10% deterioration in the Global Health Status/Quality of Life (GHS/QoL) Scale Score of the European Organization for Research and Treatment of Cancer's Core Quality of Life Questionnaire (EORTC QLQ-C30), change from baseline in the GHS/QoL Scale Score of the EORTC QLQ-C30
**MONARCH-2 NCT02107703**	Study Phase: Phase 3 Allocation: Randomized	Women with HR+, HER2-advanced breast cancer who had progressed while	Australia, Belgium, Canada, Denmark, Finland, France, Germany, Greece,	Abemaciclib + fulvestrant *vs.* placebo + fulvestrant	PFS, OS, ORR, DOR, DCR, CBR, change from baseline in pain and symptom burden assessment using the Modified

	Masking: Double (participant, investigator)	receiving neoadjuvant or adjuvant endocrine therapy (ET), ≤ 12 months from the end of adjuvant ET, or while receiving first-line ET for metastatic disease	Italy, Japan, Republic of Korea, Mexico, Poland, Puerto Rico, Romania, Russian Federation, Spain, Switzerland, Taiwan, United States		Brief Pain Inventory-Short Form (mBPI-sf), Pharmacokinetics, change from baseline in health status using the EuroQol 5-Dimension 5 Level (EQ-5D 5L), change from baseline to short term follow up in quality of life using the European Organization for Research and Treatment of Cancer Quality of Life Questionnaire-Core 30 (EORTC QLQ-C30), change from baseline to short term follow up in quality of life using the EORTC QLQ-BR23 (Breast) Questionnaire
**MONARCH-3 NCT02246621**	Study Phase: Phase 3 Allocation: Randomized Masking: Double (participant,	Postmenopausal women with hormone receptor-positive, HER2-negative locoregionally recurrent or	Australia, Austria, Belgium, Canada, France, Germany, Greece, Israel, Italy, Japan, Republic of Korea, Mexico,	Nonsteroidal aromatase inhibitors (anastrozole or letrozole) plus abemaciclib *vs.*	PFS, OS, ORR, DOR, DCR, CBR, change from baseline to end of study in symptom burden on the European Organization for Research and Treatment of Cancer Quality of Life

	care provider)	metastatic breast cancer with no prior systemic therapy	Netherlands, New Zealand, Puerto Rico, Russian Federation, Slovakia, Spain, Sweden, Taiwan, Turkey, United Kingdom, United States	placebo plus NSAI	Questionnaire-Core 30 (EORTC QLQ-C30) functional scale scores, change from baseline to end of study in symptom burden on the EORTC QLQ-C30 symptom scale scores, change from baseline to end of study in symptom burden on the EORTC QLQ-Breast23 Questionnaire, change from baseline to end of study in health status on the EuroQuol 5-Dimension 5 Level (EuroQol-5D 5L) index value, change from baseline to end of study in health status on the EuroQol-5D 5L Visual Analog Scale (VAS) scores scale Pharmacokinetics
**MONARCHplus NCT02763566**	Study Phase: Phase 3 Allocation: Randomized	Postmenopausal women with HR-positive, HER2-negative ABC with no	Brazil, China, India, South Africa	CohortA: Abemaciclib + NSAI *vs.* placebo + NSAI	PFS, OS, ORR, DOR, DCR, CBR, change from randomization in symptom burden on the European Organization for

	Masking: Double (participant, investigator)	prior systemic therapy in an advanced setting (cohort A) or progression on prior ET (cohort B)		CohortB: Abemaciclib + fulvestrant *vs.* placebo+fulvestrant	Research and Treatment of Cancer Quality of Life Questionnaire-C30 (EORTC QLQ-C30), Pharmacokinetics
**NCCH1607/PATHWAY NCT03423199**	Study Phase: Phase 3 Allocation: Randomized Masking: Quadruple (participant, care provider, investigator, outcomes assessor)	Hormone receptor (HR)-positive, HER2-negative advanced or metastatic breast cancer patients, regardless of menopausal status	Japan, Republic of Korea, Singapore, Taiwan	Palbociclib with tamoxifen (with or without goserelin) *vs.* placebo with tamoxifen (with or without goserelin)	PFS, OS, ORR, DOR, CBR, change from baseline between treatment comparison in European Organization for Research and Treatment of Cancer Quality of Life Questionnaire (EORTC QLQ-C30) Functional Scale Scores, change from baseline between treatment comparison in European Organization for Research and Treatment of Cancer Breast Cancer Module (EORTC QLQ BR23) Functional Scale Scores, Trough plasma concentrations of palbociclib, Trough plasma concentrations of tamoxifen/4-

					hydroxytamoxifen/N-desmethyltamoxifen/endoxifen, TEAE
**PALOMA-1 NCT00721409**	Study Phase: Phase 1/2<br>Allocation: Randomized<br>Masking: None (open-label)	First-line treatment of ER-positive, HER2-negative advanced breast cancer in postmenopausal women	Canada, France, Germany, Hungary, Ireland, Italy, Republic of Korea, Russian Federation, South Africa, Spain, Ukraine, United States	Palbociclib + letrozole vs. letrozole	TEAE at Phase 1, Treatment-Related Adverse Events at Phase 1, dose limiting toxicities at Phase 1, PFS at Phase 2, ORR at Phase 1, CBR at Phase 1, Pharmacokinetics at Phase 1, number of participants with increase from baseline in corrected QT (QTc) interval at Phase 1, OS at Phase 2, ORR at Phase 2, DOR at Phase 2, CBR at Phase 2, time to tumor progression (TTP) at Phase 2, change from baseline in Modified Brief Pain Inventory in Pain Severity Scale (mBPI-sf) Questionnaire at Phase 2, change from baseline in Modified Brief Pain Inventory in Pain Interference Scale

					(mBPI-sf) Questionnaire at Phase 2, presence or absence of tumor tissue biomarkers at Phase 2 [p16/INK4A, CCND1, Ki67, Tumor Retinoblastoma (RB) and CyclinD1], summary of copy number for CCND1 (CCND1/CEP11) and p16/INK4A (p16/CEP9) at Phase 2, percentage of participants with tumor expression of CYP19A1 and CCND1 Genotypes at Phase 2, number of participants with TEAEs (All Causalities) at Phase 2, number of participants with Treatment-Related Adverse Events at Phase 2
**PALOMA-2 NCT01740427**	Study Phase: Phase 3<br>Allocation: Randomized<br>Masking: Quadruple	Postmenopausal women with ER(+)/HER2(-) advanced breast cancer who have not	Australia, Belgium, Canada, Czechia, France, Germany, Hungary, Ireland, Italy, Japan, Republic	Palbociclib plus letrozole vs. placebo plus letrozole	PFS, ORR, DOR, DC/CBR, PFS by tumor tissue biomarkers status, including genes (e.g., copy numbers of CCND1, CDKN2A), proteins (e.g., Ki67,

(participant, care provider, investigator, outcomes assessor)	received prior systemic anticancer therapies for their advanced/metastatic disease.	of Korea, Poland, Russian Federation, Spain, Taiwan, Ukraine, United Kingdom, United States		pRb), and RNA expression (e.g., cdk4, cdk6), corrected QT interval (QTc) time-matched change from baseline on cycle 1 day 14, percentage of Participants With Corrected QT Interval (QTc), observed plasma trough concentration (C_{trough}) at steady-state, change from baseline between treatment comparison in Euro Quality of Life (EQ-5D) Index, change from baseline between treatment comparison in Functional Assessment of Cancer Therapy -Breast (FACT-B), TEAE, OS, Survival probability at 1 year, 2 year and 3 year, number of participants with laboratory abnormalities by maximum Common Terminology Criteria for Adverse Events (CTCAE)

					Grade
**PALOMA-3 NCT01942135**	Study Phase: Phase 3 Allocation: Randomized Masking: Triple (participant, care provider, investigator)	Women with HR+, HER2 negative metastatic breast cancer whose disease has progressed after prior endocrine therapy	Australia, Belgium, Canada, Germany, Ireland, Italy, Japan, Republic of Korea, Netherlands, Portugal, Romania, Russian Federation, Taiwan, Turkey, Ukraine, United Kingdom, United States	Palbociclib in combination with fulvestrant (with or without goserelin) vs. placebo with fulvestrant (with or without goserelin)	PFS, OS, survival probabilities at year 1, year 2, and year 3, ORR, DOR, CBR, Pharmacokinetics, change from baseline between treatment comparison in European Organization for Research and Treatment of Cancer Quality of Life Questionnaire (EORTC QLQ-C30) Functional Scale Scores, change from baseline between treatment comparison in EORTC QLQ-C30 Symptom Scale Scores, change from baseline between treatment comparison in European Organization for Research and Treatment of Cancer Breast Cancer Module (EORTC QLQ BR23) Functional Scale Scores, change from baseline between

					treatment comparison in EORTC QLQ BR23 Symptom Scale Scores, change from baseline between treatment comparison in EuroQoL 5D (EQ-5D)- Health Index Scores, change from baseline between treatment comparison in EQ-5D Visual Analog Scale (VAS) Scores Scale, time to deterioration, TEAE, participants with shifts from CTCAE Grade ≤2 at baseline to CTCAE Grade 3 or 4 postbaseline for hematology results, participants with shifts from CTCAE Grade ≤2 at baseline to CTCAE Grade 3 or 4 Postbaseline for chemistry results
**PALOMA-4 NCT02297438**	Study Phase: Phase 3 Allocation:	Asian postmenopausal women with	China, Hong Kong, Singapore, Taiwan, Thailand	Palbociclib plus letrozole vs. placebo plus	PFS, ORR, DOR, DC, CBR, OS, 1-year, 2-year, and 3-year survival probability, TEAE,

Randomized Masking: Quadruple (participant, care provider, investigator, outcomes assessor)	ER(+)/HER2(-) advanced breast cancer who have not received prior systemic anticancer therapies for their advanced/metastatic disease.		letrozole	number of participants with postbaseline laboratory abnormalities of Common Terminology Criteria for Adverse Events (CTCAE) Grade 3 or 4- Hematology/Chemistry, trough plasma concentration of palbociclib, model estimated mean change from baseline in Euro Quality of Life 5-Dimension Scale (EQ-5D) Index Scores, model estimated mean change from baseline in Euro Quality of Life (EQ) Visual Analog Scale (VAS) Scores, model estimated mean changes from baseline in Functional Assessment of Cancer Therapy - Breast (FACT-B) Total Score, median baseline percent (%) positive cells for Ki67, number of participants with detection in

estrogen receptor

ORR: objective response rate; DOR: duration of objective response; CBR: clinical benefit rate; SAE: serious adverse event; AE: adverse event; TEAE: treatment-emergent adverse events; DCR: disease control rate; TTR: Time to Response; NSAI: Nonsteroidal aromatase inhibitors; DC: disease control; OR: objective response; PFS: progression-free survival; OS: overall survival

Supplementary Table 3. Ranking CDK4/6 inhibitor by the probability of achieving the highest clinical benefit in terms of PFS.

Medication (contrast to placebo)	**HR (95% CI) -random effect model**	**P-score -random effect**
1. lerociclib	0.46 (0.32-0.66)	0.8311
2. abemaciclib	0.51 (0.45-0.58)	0.7163
3. dalpiciclib	0.51 (0.38-0.68)	0.6817
4. ribociclib	0.57 (0.51-0.64)	0.4186
5. palbociclib	0.58 (0.53-0.65)	0.3524

HR: hazard ratio; CI: confidence interval.

Supplementary Table 4. Ranking CDK4/6 inhibitor by the probability of achieving the highest clinical benefit in terms of PFS: subgroup analysis by lines of therapy.

**1st line**		**≧2nd lines**	
Medication	P-score -random effect	***Medication***	P-score -random effect
1. dalpiciclib	0.7805	*1. lerociclib*	0.7652
2. abemaciclib	0.7541	*2. abemaciclib*	0.7306
3. ribociclib	0.6057	*3. palbociclib*	0.5205
4. palbociclib	0.3597	*4. ribociclib*	0.4829

Supplementary Table 5. Ranking CDK4/6 inhibitor by the probability of achieving the highest clinical benefit in terms of PFS: subgroup analysis by *PIK3CA* status.

***PIK3CA* wild-type**		***PIK3CA* mutant**	
Medication	P-score<br>-random effect	***Medication***	P-score<br>-random effect
1. abemaciclib	0.8113	*1. palbociclib*	0.7661
2. palbociclib	0.6269	*2. abemaciclib*	0.6649
3. ribociclib	0.5595	*3. ribociclib*	0.5612

Supplementary Table 6. Ranking CDK4/6 inhibitor by the probability of achieving the highest clinical benefit in terms of OS.

Medication (contrast to placebo)	**HR (95% CI) -random effect model**	**P-score -random effect**
1. ribociclib	0.76 (0.68-0.85)	0.8683
2. abemaciclib	0.79 (0.68-0.92)	0.7128
3. palbociclib	0.87 (0.76-0.99)	0.4129

HR: hazard ratio; CI: confidence interval.

Supplementary Table 7. Ranking CDK4/6 inhibitor by the probability of achieving the highest clinical benefit in terms of OS: subgroup analysis by lines of therapy.

**1st line**		**≧2nd lines**	
Medication	P-score -random effect	***Medication***	P-score -random effect
1. ribociclib	0.8995	*1. abemaciclib*	0.7055
2. abemaciclib	0.6942	*2. ribociclib*	0.6376
3. palbociclib	0.3255	*3. palbociclib*	0.6292

Supplementary Table 8. The analysis of gene alteration tendencies among the alterations in *PIK3CA*, *TP53*, *ESR1*, *RB1*, *CCNE1*, *CCND1*, *BRCA1*, and *BRCA2*. The analysis evaluated 28 pairwise associations among the eight gene alteration tracks in the OncoPrint. The gene dataset was sourced from the Metastatic Breast Cancer (MSK, Cancer Discovery 2022) database, comprising 1,116 patients with HR+/HER2- metastatic breast cancer. A p-value and q-value < 0.05 indicate statistical significance for co-occurrence or mutual exclusivity.

A	B	Neither	A Not B	B Not A	Both	Log2 Odds Ratio	p-Value	q-Value	Tendency
ESR1	***CCND1***	823	187	228	127	1.294	*<0.001*	*<0.001*	Co-occurrence
TP53	***RB1***	950	352	31	32	1.478	*<0.001*	*0.002*	Co-occurrence
TP53	***ESR1***	734	317	247	67	-0.671	*0.002*	*0.02*	Mutual exclusivity
RB1	***CCND1***	955	55	347	8	-1.321	0.012	0.085	Mutual exclusivity
RB1	***BRCA2***	1245	57	57	6	1.201	0.066	0.277	Co-occurrence
PIK3CA	***CCND1***	599	411	230	125	-0.336	0.077	0.277	Mutual exclusivity
CCNE1	***BRCA1***	1313	16	34	2	2.271	0.079	0.277	Co-occurrence
TP53	***BRCA2***	942	360	39	24	0.687	0.085	0.277	Co-occurrence
TP53	***BRCA1***	960	369	21	15	0.894	0.089	0.277	Co-occurrence
PIK3CA	***RB1***	797	505	32	31	0.612	0.113	0.316	Co-occurrence
TP53	***CCNE1***	971	376	10	8	1.047	0.183	0.449	Co-occurrence
TP53	***CCND1***	716	294	265	90	-0.274	0.192	0.449	Mutual exclusivity
ESR1	***RB1***	998	304	53	10	-0.691	0.219	0.458	Mutual exclusivity
BRCA1	***BRCA2***	1269	33	60	3	0.943	0.229	0.458	Co-occurrence
CCND1	***BRCA1***	980	349	30	6	-0.832	0.249	0.464	Mutual exclusivity
ESR1	***CCNE1***	1039	308	12	6	0.754	0.272	0.476	Co-occurrence
CCND1	***BRCA2***	960	342	50	13	-0.454	0.379	0.624	Mutual exclusivity

PIK3CA	***TP53***	589	392	240	144	-0.15	0.423	0.63	Mutual exclusivity
ESR1	***BRCA1***	1021	308	30	6	-0.593	0.427	0.63	Mutual exclusivity
CCNE1	***BRCA2***	1285	17	62	1	0.286	0.575	0.798	Co-occurrence
PIK3CA	***ESR1***	634	417	195	119	-0.108	0.599	0.798	Mutual exclusivity
ESR1	***BRCA2***	1004	298	47	16	0.198	0.646	0.823	Co-occurrence
CCNE1	***CCND1***	997	13	350	5	0.132	0.793	0.944	Co-occurrence
PIK3CA	***CCNE1***	817	530	12	6	-0.376	0.809	0.944	Mutual exclusivity
PIK3CA	***BRCA1***	808	521	21	15	0.148	0.863	0.964	Co-occurrence
PIK3CA	***BRCA2***	790	512	39	24	-0.075	0.895	0.964	Mutual exclusivity
RB1	***CCNE1***	1284	63	18	0	<-3	1	1	Mutual exclusivity
RB1	***BRCA1***	1267	62	35	1	-0.776	1	1	Mutual exclusivity
